# Predicting cell-penetrating peptides using machine learning algorithms and navigating in their chemical space

**DOI:** 10.1038/s41598-021-87134-w

**Published:** 2021-04-07

**Authors:** Ewerton Cristhian Lima de Oliveira, Kauê Santana, Luiz Josino, Anderson Henrique Lima e Lima, Claudomiro de Souza de Sales Júnior

**Affiliations:** 1 Institute of Technology, Federal University of Pará, Belém, Pará 66075-110 Brazil; 2 Institute of Biodiversity, Federal University of Western Pará, Vera Paz street, s/n Salé, Santarém, Pará 68040-255 Brazil; 3 Laboratório de Planejamento e Desenvolvimento de Fármacos, Instituto de Ciências Exatas e Naturais, Universidade Federal do Pará, Belém, Pará 66075-110 Brazil

**Keywords:** Software, Computational biology and bioinformatics

## Abstract

Cell-penetrating peptides (CPPs) are naturally able to cross the lipid bilayer membrane that protects cells. These peptides share common structural and physicochemical properties and show different pharmaceutical applications, among which drug delivery is the most important. Due to their ability to cross the membranes by pulling high-molecular-weight polar molecules, they are termed Trojan horses. In this study, we proposed a machine learning (ML)-based framework named BChemRF-CPPred (***b****eyond* ***chem****ical* ***r****ules-based* ***f****ramework for* ***CPP pred****iction*) that uses an artificial neural network, a support vector machine, and a Gaussian process classifier to differentiate CPPs from non-CPPs, using structure- and sequence-based descriptors extracted from PDB and FASTA formats. The performance of our algorithm was evaluated by tenfold cross-validation and compared with those of previously reported prediction tools using an independent dataset. The BChemRF-CPPred satisfactorily identified CPP-like structures using natural and synthetic modified peptide libraries and also obtained better performance than those of previously reported ML-based algorithms, reaching the independent test accuracy of 90.66% (AUC = 0.9365) for PDB, and an accuracy of 86.5% (AUC = 0.9216) for FASTA input. Moreover, our analyses of the CPP chemical space demonstrated that these peptides break some molecular rules related to the prediction of permeability of therapeutic molecules in cell membranes. This is the first comprehensive analysis to predict synthetic and natural CPP structures and to evaluate their chemical space using an ML-based framework. Our algorithm is freely available for academic use at http://comptools.linc.ufpa.br/BChemRF-CPPred.

## Introduction

Peptides are a structurally diverse class of bioactive molecules with several physicochemical and structural properties^1,2^. Naturally derived peptides have numerous pharmaceutical applications, such as acting selectively against pathogens^3,4^, and human targets^5,6^; and as cargo and delivery vehicles of covalently bound bioactive molecules, such as drugs, small-interfering RNAs (siRNAs), plasmids, and nanoparticles^7–10^. Additionally, the recent advances in peptide synthesis have led to increased use in the pharmaceutical industry, because of their improved potency, specificity against molecular targets, and permeability to cell membranes^11,12^.

The cell membrane is considered the main obstacle for therapeutic molecules to reach their active sites in cells. The selective control of the permeability of molecules through the cell membrane regulates passive diffusion and active transport to the intracellular medium impairing the entrance of some therapeutic compounds^13^. Cell-penetrating peptides (CPPs) can naturally cross the lipid bilayer membrane that protects the cells. These peptides share common structural and physicochemical features: they contain a sequence length between 5 and 42 amino acids, (2) they are soluble in water and partially hydrophobic, (3) they are often cationic (positive charge at physiological pH) or amphipathic, and (4) they are rich in the arginine and lysine residues^14,15^. CPPs possess a wide range of biological activities, such as antiviral^16,17^, antifungal^18^, and antibacterial activities^19,20^, thus showing potential in pharmaceutical applications, but the main category has being drug delivery systems^21–23^, and because they can cross the membranes pulling high molecular weight polar molecules, they are termed Trojan horses^10,24–27^. The Trojan horse refers to the mythical story about a stratagem of the ancient Greeks used to enter the fortified walls of Troy city to win the war against their historical enemies. The metaphor of a Trojan horse is applied in drug delivery strategies that aim to access securely a target inside the cells ‘wearing’ the bioactive compound using the CPPs as ‘protected disguise’ to penetrate into cell membranes^28^. Due to their high structural complexity and chemical versatility, different studies have focus efforts on the prediction of their mechanisms and efficiency of transport and penetration into the cell membranes^29–37^. Different mechanisms for uptake into the cell have been described for CPPs, including endocytosis, membrane lysis, membrane translocation by passive diffusion, translocation across endosomal membrane, degradation and/or recycling of endosomal, and aggregation leading to pore formation^25,38–40^.

Computational approaches, such as cheminformatics^41–43^, artificial intelligence^44–48^, probabilistic models^49,50^, and molecular modeling tools^51–54^ have been applied to facilitate high-throughput screening of new bioactive molecules. Machine learning (ML) methods have proved as an efficient approach to select, filter, and predict compounds properties giving accurate predictions, improving decisions regarding drug development, and shedding light on the pharmacokinetics and pharmacodynamics properties of these compounds^55–59^.

Recently, many researchers have focused on ML techniques to predict CPPs using sequence-based descriptors. Fu et al. (2019) applied support vector machine (SVM) with an RBF kernel to predict CPPs based on the amino acid composition of the sequences^60^. Similarly, Qiang et al. (2018) developed a tool named CPPred-FL that applies 45 trained random forest (RF) models using 19 descriptors related to amino acid composition, specific-position information, and physicochemical properties to predict CPPs^61^. Pandey et al. (2018) proposed a framework named KELM-CPPpred using kernelized extreme learning machine (ELM) that also applied amino acid composition of the sequences^29^.

In contrast, other studies combined sequence- and structure-based descriptors and achieved improved accuracy for screening CPPs. Manavalan et al. (2018) proposed a framework based on the features of amino acid composition and physicochemical properties using RF, SVM, ERT, and k-nearest neighbor (K-NN) to predict CPPs and non-CPPs^31^. Kumar et al. (2018) proposed the CellPPD-Mod, a computational tool that uses RF to predict CPPs from non-CPPs with lengths up to 25 residues, based on amino acid composition, 48 two-dimensional (2D)/three-dimensional (3D) molecular descriptors, and molecular fingerprints^34^. However, to the best of our knowledge, no previous study evaluated the influence of physicochemical and structural properties related to permeability in biological membranes using ML-based tools to predict CPPs structures and to investigate their chemical space.

## Results and discussion

In this study, we proposed the BChemRF-CPPred, an ML-based framework that applies an artificial neural network, a support vector machine, and a Gaussian process classifier to predict CPPs structures using structure-based descriptors (physicochemical and structural properties) related to the permeability of these structures into the cell membranes and the presence of polar charged groups^62–64^; and sequence-based descriptors obtained from the primary structure of the peptides. We compared the overall performance of our proposed framework with four state-of-the-art methods and validated the results using statistical analysis to evaluate the feature correlation, spatial distribution of peptide properties, and information gain of the applied properties. Moreover, we evaluated the chemical space of these peptides using statistical methods and correlated them with previous conventional filters applied to predict cell permeability.

### Cell-penetrating peptides present chemical space beyond the intervals dictated by conventional filters

Over the years, the pharmaceutical industry and medicinal chemists have determined principles for drug-like molecules and predicted their permeability in biological membranes^42,65–67^. Efficiency in membrane permeation has been pointed out as a crucial factor for the bioavailability of therapeutic molecules^68^. Different studies have demonstrated that physicochemical and structural properties of peptides are outside the traditional chemical space present in the approved drugs^69–71^. These findings have helped to drive the design and discovery of novel compounds that occupy the chemical space beyond the intervals dictated by the Lipinski rules-of-five (RO5) filter^42,64^.

The structural flexibility of compounds might influence their translocation in the mobile aqueous phase due to the reduced entropic environment of the cell membranes^62^. In contrast, the flexibility might increase the entropic barriers of molecules, impairing or decreasing their affinity with the molecular targets, when compared with their restrained and cyclic counterparts^63,72^. High molecular weight (MW), topological polar surface area (tPSA), and the number of rotatable bonds (NRB) have been reported as the main limitations of some molecules to cross the cell membrane by passive permeation due to the increased molecular volume, and complexation with water molecules^62,65^.

Comparing our results with those of clinically approved peptides for oral use, we identified that CPPs have an increased MW (331.48–3750.51) and tPSA (101.29–1782.83)^71^. Due to the different reported mechanisms of cell membrane penetration, these discrepancies could be related to other mechanisms not related to passive diffusion, such as pore formation or endocytosis representative of TP-10 and caveolin-1, respectively^73–75^. The MW and tPSA values found for the analyzed CPP structures are better correlated with values previously found for linear and cyclic pentapeptides^69^.

The tPSA is correlated with the H-bond pattern of an investigated molecule in an aqueous solvent^76^. The CPPs structures investigated in our study exceeded the maximum values for clinically approved molecules, reaching values equal to 1782.83 Å^2^. Permeability into the cell membranes is typically limited when tPSA exceeds 140 Å^2^. However, studies have demonstrated that chameleonic molecules and macrocyclic peptides permeable to the biological membranes exceed these values^66,77^. Some peptides permeable to the lipid bilayer membrane using passive diffusion, such as pAntp have been described with some chameleon-like properties, i.e. can change their conformation by exposing polar groups in an aqueous medium*,* but hiding them when traversing the cell membranes^78^. It is interesting to note that a previous study identified that highly permeable peptoids and peptides showed an average tPSA value of 335.30 Å^2^ and 358.80 Å^2^, respectively^79^. These results are different from those found for our analyzed CPP datasets that showed an average of 852.42 Å^2^.

It has been demonstrated that flexible molecules can form intrachain H-bond interactions, thus adaptively reducing their polarity surface and improving the permeation into the cell membranes^80^. In this study, the molecular flexibility and complexity were measured by two structural properties: the fraction of sp^3^-hybridized carbon atoms (Fsp^3^) and the number of rotatable bonds (NRB) (Table S1). Recently, Doak et al. (2014) extended the NRB value previously found by Veber rules and indicated that bioavailable drugs present NRB < 20^62,64^. Our analyses demonstrated that CPPs exceed the maximum value of molecular properties indicated for oral drugs and peptides^69,71^, showing a range from 9 to 137 (90th percentile equal to 98.60, Table S2). Regarding Fsp^3^, studies have demonstrated that it is an important molecular property related to both solubilities in the aqueous phase and melting point^63^. We identified that for CPPs, Fsp^3^ is not inferior to 0.37 and does not exceed 0.84 (90th percentile 0.784). Our results are consistent with orally available peptides that showed 90th percentile equal to 0.79^71^.

Regarding lipophilicity, we investigated this property using the 1-octanol/water partition coefficient (cLogP). High cLogP values are related to the high lipophilicity of the molecule, thus indicating a better membrane cell penetration. Doak et al. (2014) indicated that cLogP in available drugs varies in the range − 2 ≤ cLogP ≤ 10. Here, we found that the evaluated CPP dataset showed − 42.12 ≤ cLogP ≤ 2.97, which is consistent with previous findings for cyclic pentapeptides.

Hydrogen bond acceptors (HBA) and hydrogen bond donors (HBD) are relevant factors for cell permeability by RO5. Our results showed a consistent correlation with previous values found for linear and cyclic pentapeptides^69^. However, regarding HBD, CPPs showed a high discrepancy related to clinically approved drugs (see Table S1)^64^.

Regarding the number of aromatic rings (NAR) our study found a 95th percentile equal to 6, with maximum and minimum limits equal to 0 and 10, respectively. Despite previous studies no reported its value in the analyzes of the chemical space of peptides^69,71^, it is a relevant structural property related to the lipophilicity of compounds, and studies have demonstrated that the addition of an aromatic ring usually results in a statistically significant increase in the clogP value of the compound^81^. This value represents a statistically significant component of a molecule’s overall properties in the context of the membrane permeability (the average NAR in oral drugs is equal to 1.6)^81^. Furthermore, this property is present in some molecular filters that analyze the permeability and drug-likeness of compounds^82,83^.

Analyzing the 90th percentile calculated for the physicochemical properties, the results reinforce that the CPPs structures are beyond the previously established chemical rules. Thus, indicating that these molecular intervals applied to predict the permeability of peptides into the cell membrane by passive diffusion, could not be correctly applied for this class of peptides, consequently, leading to recognize bias and hindering of the computer-aided design of CPP-like structures. The histograms of these structure-based descriptor distributions of all analyzed CPPs structures are shown in support information Figure S1.

### BChemRF-CPPred performed better using an optimized combination of structure- and sequence-based descriptors

In the present study, we investigated two class of molecular descriptors: (1) the structure-based descriptors that include structural and physicochemical properties related to the permeation of molecules into the biological membranes which are obtained from the molecular structures of peptides—MW, tPSA, Fsp^3^, cLogP, HBA, HBD, NAR, NRB, and net charge (NetC)—^64,84^, as well as, some properties related to the polar charged groups—primary amine groups (NPA), number of guanidine groups (NG), and number of negatively charged amino acid groups (NNCAA)—that could influence in their permeability; and, (2) sequence-based descriptors, i.e., information calculated from the primary structure of the peptide—amino acid composition (AAC), pseudo-amino acid composition (PseAAC), and dipeptide composition (DPC)^29,33,85^. Regarding the sequence-based descriptors, two amino acid compositions related to arginine (f[Arg]) and lysine (f[Lys]) fractions were analyzed in our algorithm due to their relevance in the characterization of this class of peptides^14,15^. We also analyzed two other descriptors in the ML-based framework: the DPC to evaluate the presence of motifs in the CPP sequences that are relevant to their mechanism uptake into the cell^86,87^; and the PseAAC to predict the overall peptides attributes^29,33,61^. The PseAAC is a theoretical molecular descriptor formed by a combination of discrete sequence correlation factors and twenty components of the conventional amino acid composition^88^. Our algorithm uses as input datasets both primary and tertiary structures of peptides in FASTA or PDB formats, respectively. To train the ML-based frameworks that use the tertiary structure of the peptides (PDB format), we selected two datasets, that were divided into training (600 peptide structures) obtained from curated databases and an independent test (150 structures) obtained from the literature. In contrast, to train the ML-based frameworks that used the primary structure of the peptides (FASTA format), we considered only peptides containing natural residues in the training dataset that were accounted for a total of 241 CPPs and 300 non-CPPs, and for the independent test, we considered only the natural peptides from the original dataset, which account 60 CPPs and 75 non-CPPS.

To understand the influence of structure- and sequence-based descriptors on framework performance, we first formed four FCs: FC-1 containing only sequence-based descriptors (AAC, PseAAC, and DPC); FC-2 containing only structure-based descriptors (structural and physicochemical properties); FC-3 containing the best correlated sequence-based descriptors and structure-based descriptors; and FC-4 containing an optimized selection of structure- and sequence-based descriptors according to Kendall’s correlation analysis (see Figures S2, S3, and S4): AAC, PseAAC, the 10 most-well correlated DPC, and the 9 better correlated structure-based descriptors (excluding tPSA, NRB, and HBD).

**Figure 1:**
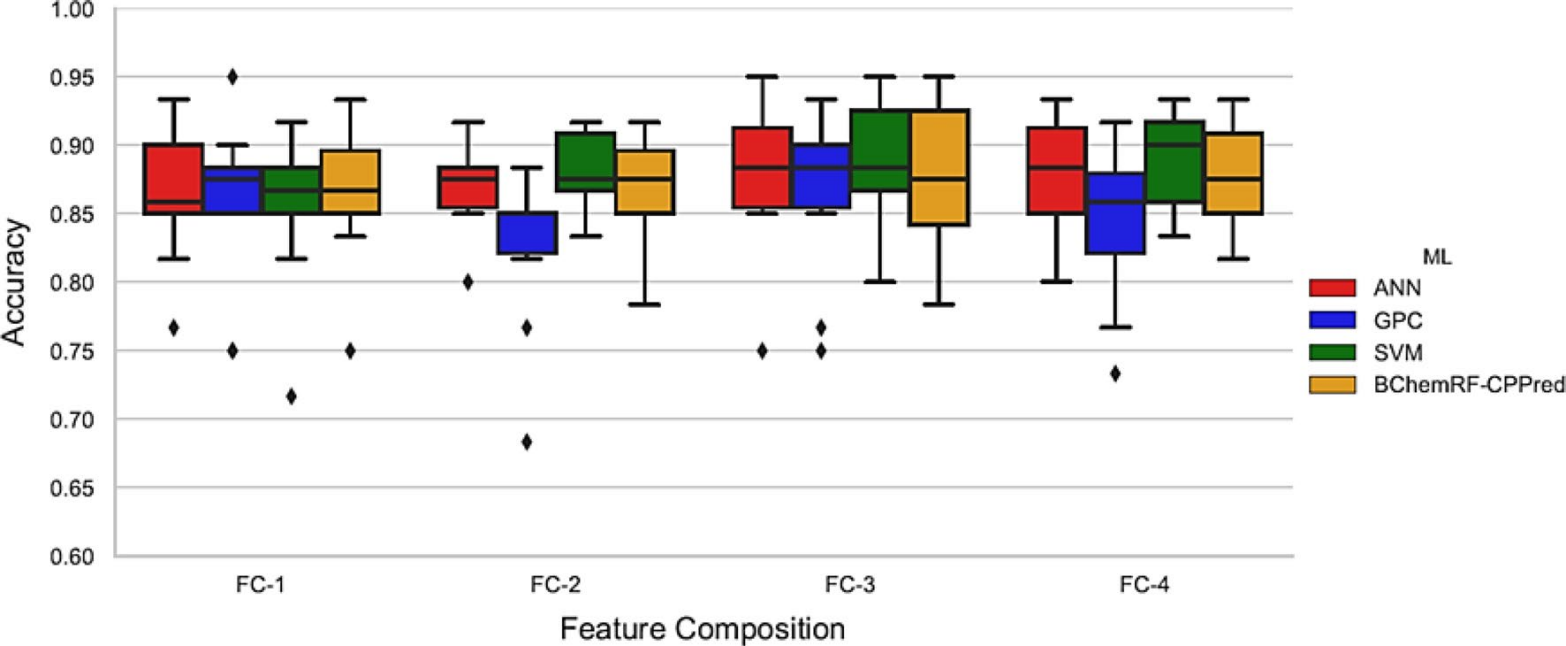
Boxplot of accuracy from tenfold cross-validation of ANN (red), GPC (blue), SVM (green), and BChemRF-CPPred (orange).

Second, we evaluated the prediction performance of the BChemRF-CPPred and its classifiers an ANN, GPC, and SVM using tenfold cross-validation in the training dataset (Fig. 1). The hyper-parameters of each classifier by FC are listed in Table S3.

In Fig. 1, we observed the performance of each estimator using tenfold cross-validation analyses. FC-1 and FC-2 reached the worst results, where BChemRF-CPPred obtained an average accuracy level of 86.5%, while their ML algorithms achieved values between 85.5 and 86.5% for the FC-1, and between 82.6 and 88% for the second one.

The framework that used FC-3 obtained an average accuracy of 87.83%, while ANN, GPC, and SVM achieved 88%, 86.5%, and 88.83%, respectively. Considering FC-4, the BChemRF-CPPred achieved an accuracy equal to 87.66%, and these classifiers obtained an average accuracy of 87.5%, 84.16, and 89%, respectively. Although the FC-3 had reached a slightly better average accuracy than FC-4, the Kruskal–Wallis H test (*p* value = 0.820) showed no statistically significant difference between the accuracies obtained by the frameworks that used these FCs. Furthermore, the framework that uses the FC-4 (43 descriptors) is less complex than those that use FC-3 (73 descriptors).

It is important to note that, although the FC-1 (containing only sequence-based descriptors) and the FC-2 (only structure-based descriptors) have shown relevant correlation to CPPs' prediction, according to Kendall's correlation analysis, these descriptors isolated do not provide enough information to predict satisfactorily the permeability of these peptides into the cell membranes. Our results showed that the optimized combination of structure- and sequence-based descriptors (FC-4) better predict natural and synthetic CPPs than other analyzed FCs.

### Evaluating the performance of BChemRF-CPPred in comparison with previous proposed computational tools

The independent test was performed with 75 CPP and 75 non-CPP structures. Among the CPPs investigated at this stage, we analyzed the 7 structures with high uptake into the cell membranes: LDP-NLS, MAP 8, synB3, ptat4, aminopeptidase, EB1, pAntpHD 40p2; and 7 peptides with no permeability to cell membranes: pAntp(4–13), motilin, vasopressin, bradykinin, scr pVec, Bax BH3, and Mut-LDP-NLS.

**Figure 2:**
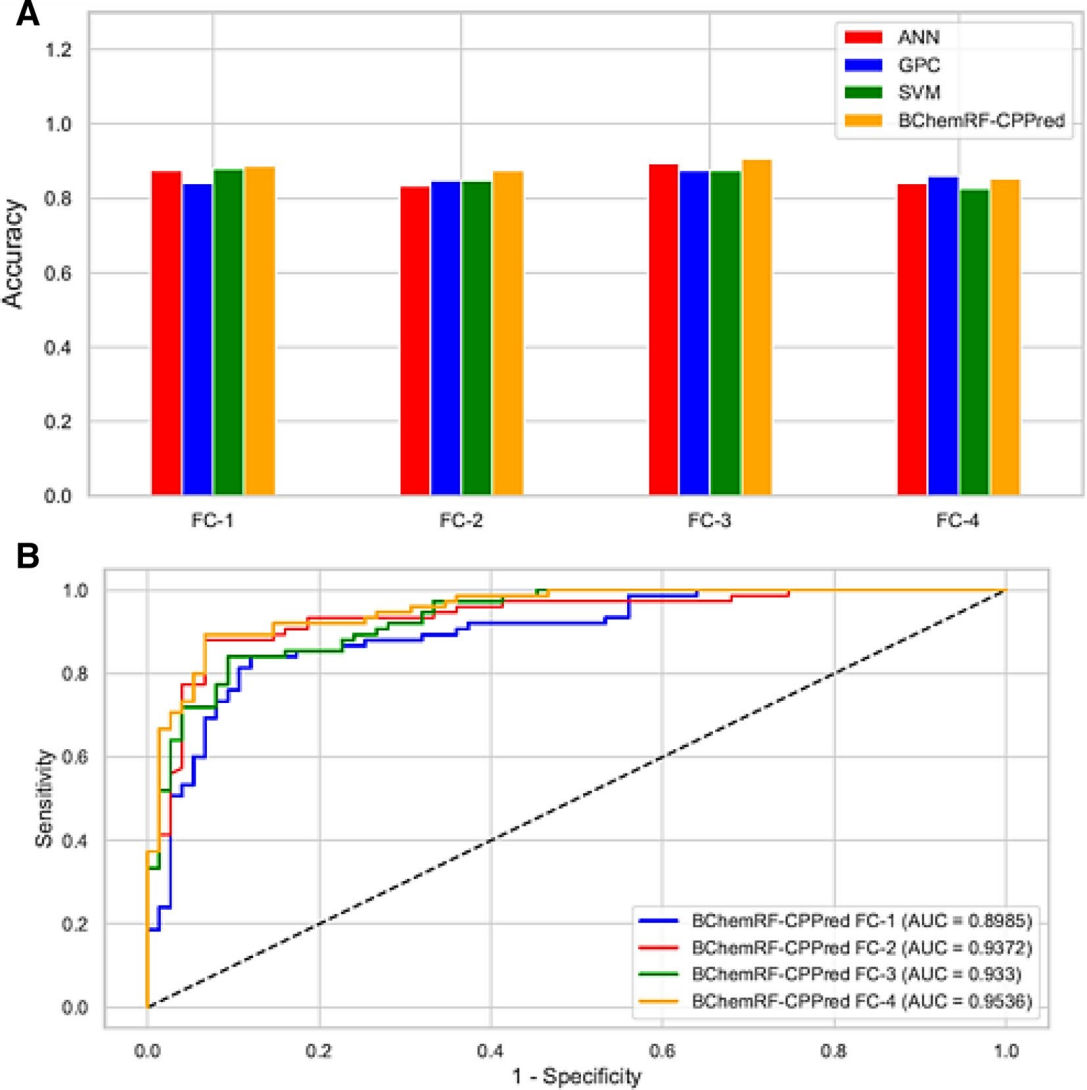
(**A**) Accuracy of ANN (red), GPC (blue), SVM (green), and BChemRF-CPPred (orange) by FCs evaluated in the independent test. (**B**) ROC curves and AUC of ML-based frameworks using the FC-1, FC-2, FC-3, and FC-4 in the independent test.

Our analyses revealed that the BChemRF-CPPred based on feature compositions with more information (FC-3 and FC-4), obtained an accuracy greater than 85%, as shown in Fig. 2A. FC-4 demonstrated 90.66% accuracy, while FC-1, FC-2, and FC-3 obtained an accuracy of 85.33%, 88.66%, and 87.33%, respectively.

The receiver operating characteristic (ROC) curves and their area under curve (AUC) metric revealed the impact of each descriptor composition in our proposed framework (Fig. 2B). Although the molecular properties have shown a satisfactory contribution in FC-2 and FC-3, reaching AUC values 0.9372 and 0.933, respectively, when compared to FC-1 that obtained an AUC value of 0.8985 and has only AAC, DPC, and PseAAC, the descriptors present in FC-4 achieved AUC value of 0.9536, providing more information for the BChemRF-CPPred to predict the cell membrane permeation of CPPs.

The behavior of the ROC curves observed in Fig. 2B corroborates previous results, since the curve associated with the FC-4 based framework (orange curve) is closer to the left corner of the graph, which indicates a higher true positive rate and a lower false-positive rate in the prediction of CPPs and non-CPPs compared with the other FCs.

Table 1 shows a detailed analysis of FC-4 in terms of accuracy, sensitivity, specificity, and Matthews correlation coefficient (MCC). These results show that the framework showed an improved ability to correctly differentiate non-CPPs from CPPs. Furthermore, the highest MCC and one of the greatest accuracies and F1-score with values of 0.813, 0.906, and 0.905, respectively, proved that BChemRF-CPPred is the best classifier among the four analyzed ones.

**Table 1:** Comparison of accuracy, sensitivity, specificity, F1-score, and MCC obtained for ANN, GPC, SVM, and BChemRF-CPPred in the independent test using FC-4.

Method	Sensitivity	Specificity	Accuracy	F1-score	MCC
ANN	0.880	0.906	0.893	0.891	0.786
GPC	0.853	0.893	0.873	0.870	0.747
SVM	0.853	0.893	0.873	0.870	0.747
BChemRF-CPPred	0.893	0.920	0.906	0.905	0.813

To compare our FC-4 based framework with state-of-the-art methods for CPP prediction, we divided this analysis into two experiments. The first one analyses our method with tools that were trained with only natural peptides, such as MLCPP^31^, CPPred-RF^33^, and SkipCPP-Pred^89^. This group was analyzed with 60 CPPs (chemically unmodified peptides) and 75 non-CPPs from the independent test dataset. The second experiment compared our framework with Kelm-CPPpred^29^, an algorithm trained with synthetic peptides (chemically modified), using the original independent dataset.

Table 2 compares the performance of previous ML-based frameworks trained and non-trained with synthetic peptides, respectively. These results show that by using an imbalanced dataset (first experiment) with only natural peptides, BChemRF-CPPred obtained an accuracy value of 89.62%, while MLCPP, CPPred-RF, and SkipCPP-Pred reached 86.66%, 68.88%, and 62.58%, respectively. Moreover, our framework obtained the highest values of F1-score and MCC when compared with other tools, which indicates that the structure-based descriptors provided more information to predict cell membrane permeability of natural peptides compared with sequence-based tools.

**Table 2:** Comparison of the performance of previous ML-based frameworks (MLCPP, CPPred-RF, and SkipCPP-Pred) and FC-4 based BChemRF-CPPred using only natural peptides from the independent dataset (1st experiment); as well as, the evaluation of the performance of Kelm-CPPpred and FC-4 based BChemRF-CPPred from all independent dataset (2nd experiment).

Method	Sensitivity	Specificity	Accuracy	F1-score	MCC
**First experiment**					
MLCPP	0.966	0.786	0.866	0.865	0.752
CPPred-RF	0.983	0.453	0.688	0.737	0.495
SkipCPP-Pred	0.966	0.520	0.625	0.753	0.525
BChemRF-CPPred	0.866	0.920	0.896	0.881	0.789
**Second experiment**					
Kelm-CPPpred	0.906	0.866	0.886	0.888	0.773
BChemRF-CPPred	0.893	0.920	0.906	0.905	0.813

The second experiment also revealed that the proposed ML-based framework achieved better outcomes in terms of accuracy, F1-score, and MCC when compared with Kelm-CPPpred, which demonstrates a high-performance prediction of CPPs by BChemRF-CPPred, including the synthetic (chemically modified) peptides containing methyl, glycyl, and other chemical groups. An accuracy of 90.66% demonstrated that the proposed framework using an optimized combination of structure- and sequence-based descriptors satisfactorily differentiated CPPs and non-CPPs from natural and synthetic origins.

### Accessing the performance of BChemRF-CPPred using FASTA as input format

**Figure 3:**
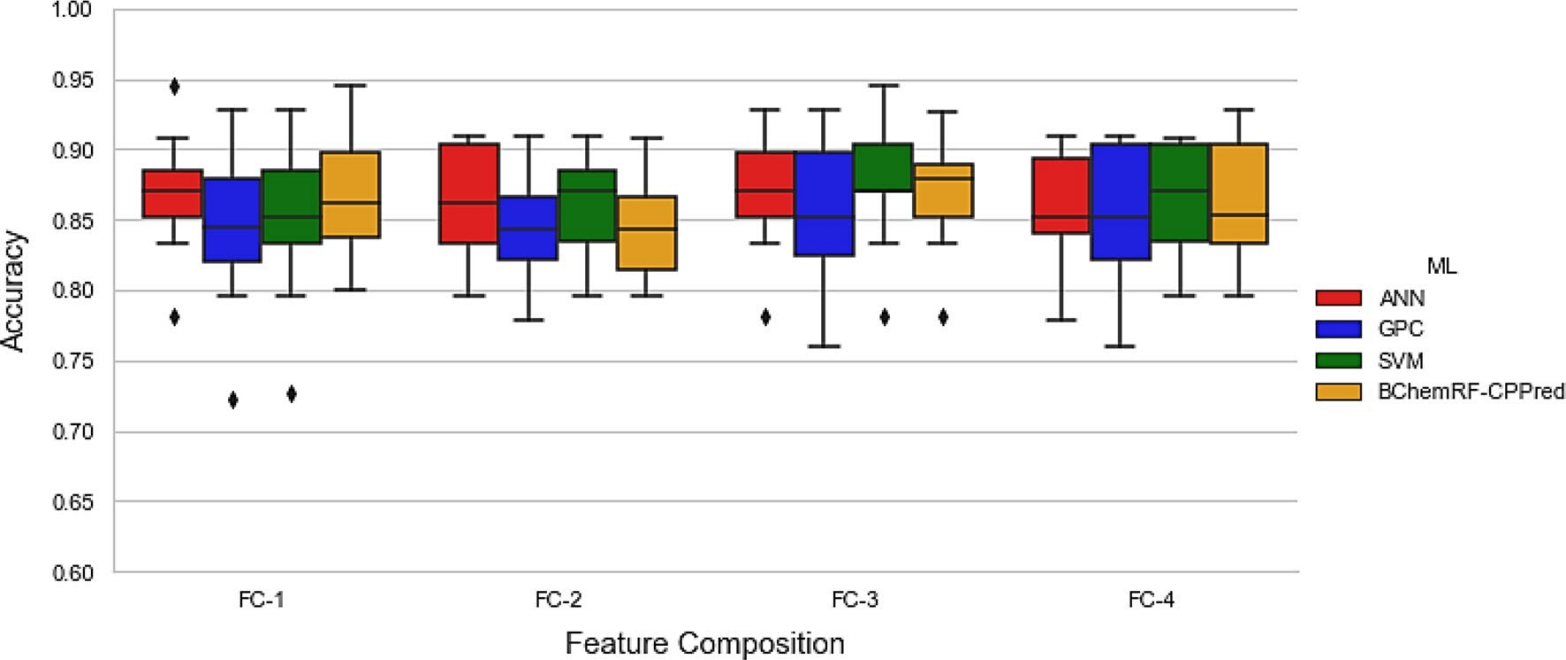
Boxplot of accuracy from tenfold cross-validation of ANN (red), GPC (blue), SVM (green), and BChemRF-CPPred (orange) using FASTA input.

To evaluate the performance of BChemRF-CPPred in the prediction of CPPs using chemical data obtained from the primary and tertiary structures, we used both FASTA and PDB formats, respectively, to calculate the four FCs using the tenfold cross-validation (Fig. 3). To train the framework, using FASTA format, we considered only peptides containing natural residues in the training dataset that were accounted for a total of 241 CPPs and 300 non-CPPs.

**Figure 4:**
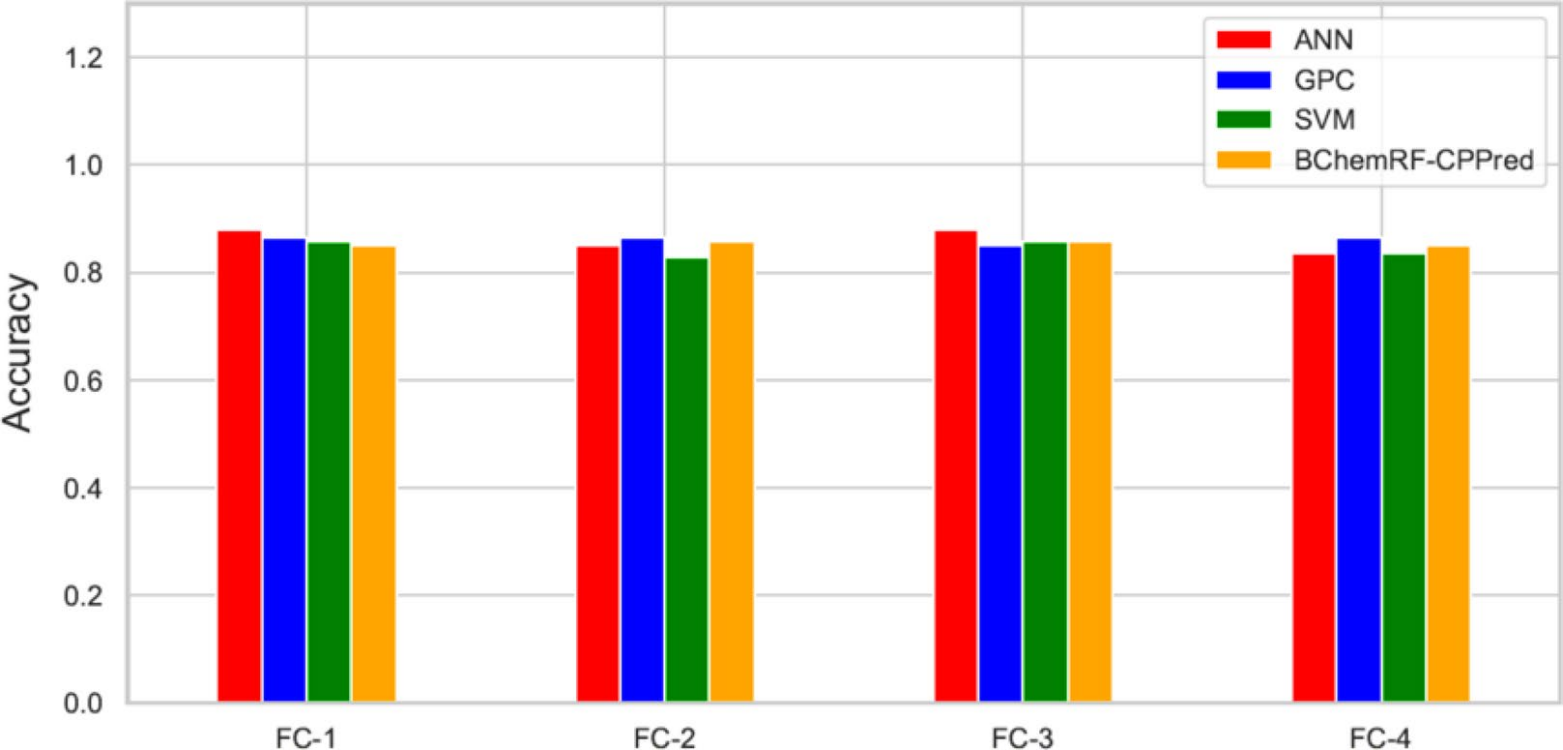
Accuracy of ANN (red), GPC (blue), SVM (green), and BChemRF-CPPred (orange) by FCs evaluated in the independent test, using FASTA input.

Figure 3 shows the performance of each classifier using the FASTA format as input according to cross-validation analyses. The framework that used the FC-3 reached the best performance with an average accuracy of 86.9%, while FC-1, FC-2, and FC-4 achieved values between 84.13 and 86.71%, respectively. When compared with the performance of BChemRF-CPPred that used as input the PDB format, the cross-validation of the framework that used FASTA as input showed a lower performance for FC-2, FC-3, and FC-4, whose accuracy values for PDB format were 86.5%, 87.83%, and 87.66%, respectively. Our analyses of the performance of BChemRF-CPPred using FC-1, composed only by sequence-based features in the independent test, revealed that the use of only natural peptides in FASTA format as input obtained an accuracy equal to 86.56%, while the FC-2, FC-3, and FC-4 achieved values of 85.07%, 85.82%, and 85.2%, respectively (Fig. 4).

Table 3 compares the performance between the FASTA-input-based framework, using all FCs (FC-1 to FC-4), and the PDB-input-based one with FC-4. This independent test uses the same testing dataset described in experiment 1 (see Table 2), which has only natural peptides.

**Table 3:** Comparison of the performance of BChemRF-CPPred frameworks that used only natural peptides in the independent test. The comparison was performed between the frameworks based on the four feature compositions (FC-1 to FC-4) that use FASTA as input with the framework based on the FC-4 that uses the PDB as input.

Input	FC	Sensitivity	Specificity	Accuracy	F1-score	MCC
FASTA	FC-1	0.813	0.906	0.865	0.842	0.726
FASTA	FC-2	0.847	0.853	0.850	0.833	0.698
FASTA	FC-3	0.813	0.893	0.858	0.834	0,711
FASTA	FC-4	0.796	0.906	0.858	0.831	0.711
PDB	FC-4	0.866	0.920	0.896	0.881	0.789

The framework that uses FC-1 obtained the best prediction results in the independent test using the FASTA format as input, i.e., the framework trained only with the sequence-based features showed higher values of accuracy, F1-score, and MCC when compared with the other frameworks that used FC-2, FC-3, and FC-4. It is important to note that both the framework based on FC-4 that uses PDB as input and the BChemRF-CPPred based on the FC-1 that uses FASTA as input performed better in the prediction of natural CPPs than previous tools CPPred-RF and SkipCPP-Pred, which reached accuracy values between 62.5 and 68.8%, F1-score values between 73.7 and 75.3%, and MCC values between 49.5 and 52.5%, respectively (Table 2).

The results also revealed that when compared the framework based on the FC-4 that uses the PDB as input with the framework based on FC-1 that uses FASTA, the Kruskal–Wallis H test (*p* value = 0.622) showed no statistically significant difference between the accuracies obtained by these two frameworks in the tenfold cross-validation. However, the PDB-based model achieved better performance in an independent test for all the metrics (Table 3).

### An optimized combination of structure- and sequence-based descriptors improved the prediction of CPPs’ structures

To analyze the influence of the sequence-based (AAC, DPC, and PseAAC) and structure-based (MW, tPSA, Fsp^3^, cLogP, HBA, HBD, NAR, NRB, NPA, NG, NetC, and NNCAA) descriptors on the performance of CPP prediction in our ML-based framework, we extracted information entropy using the extremely randomized trees (ERT) algorithm and applied principal component analyses (PCA) in all peptide datasets.

The presence of cationic residues, such as lysine and arginine, in peptides sequences, has been shown to play an important role in membrane permeation. These residues form non-covalent interactions with the anionic groups of the membrane surface. The highly basic polar groups from these residues remain protonated under physiological pH conditions, acting as hydrogen-bond donors in CPP–lipid interactions^90,91^.

**Figure 5:**
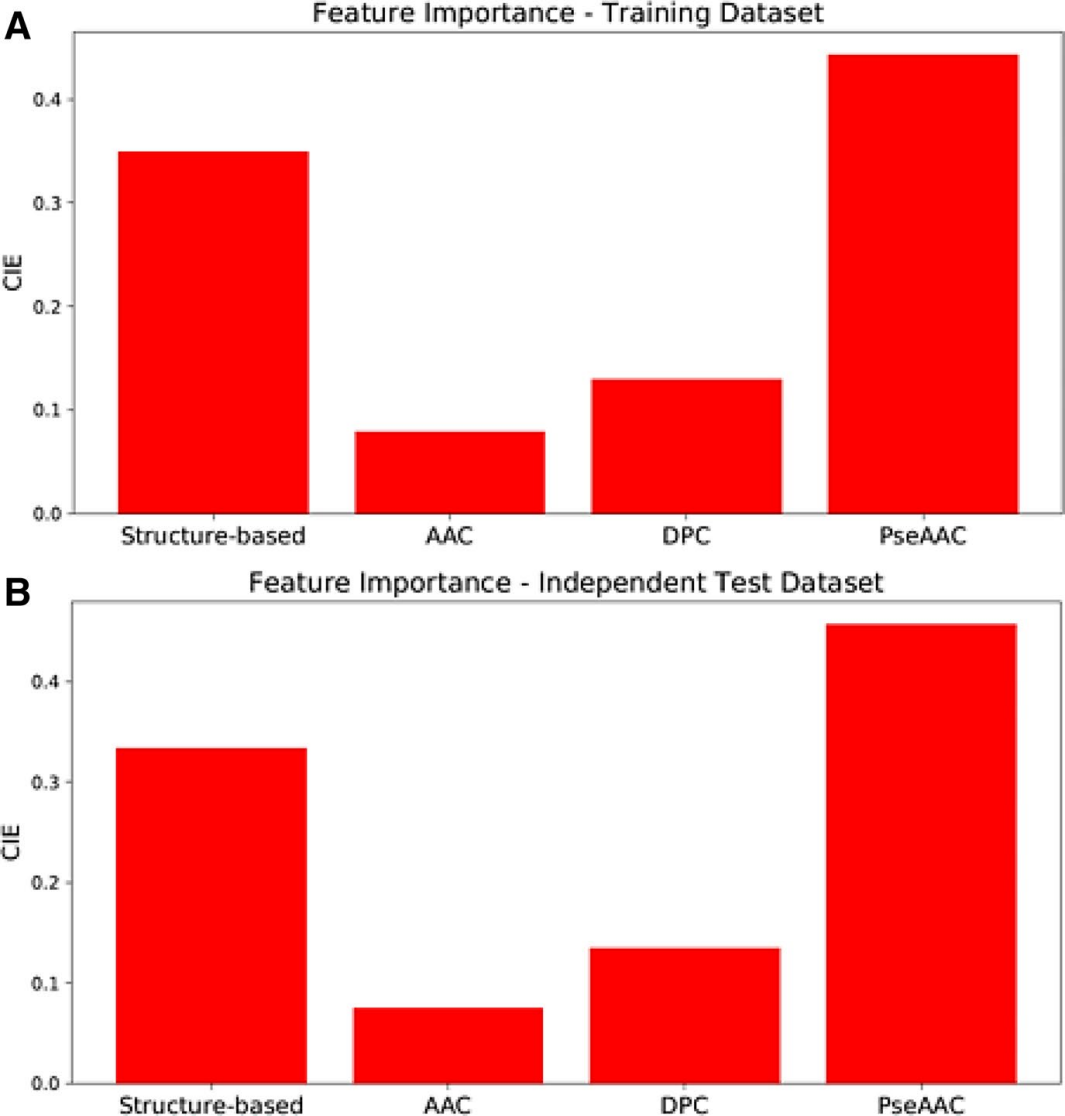
Normalized cumulative information entropy (CIE) provided by structure-based, AAC, DPC, and PseAAC descriptors, and calculated by ERT algorithm. (**A**) Training dataset; (**B**) independent test dataset.

Our study demonstrated that AAC, DPC, and PseAAC provided 0.650 and 0.664 of normalized cumulative information entropy (CIE), while the structure-based descriptors supplied 0.350 and 0.336 of CIE for training and independent test, respectively (Fig. 5).

Although the sequence-based features have several descriptors better correlated according to Kendall’s correlation when compared to structure-based descriptors, the CIE of physicochemical and structural properties showed a better contribution to CPP prediction than the AAC and DPC contributions taking together. Structural and physicochemical properties give significant information for ML algorithms, which can be verified by accuracies achieved in the independent test by the framework that used FC-4.

**Figure 6:**
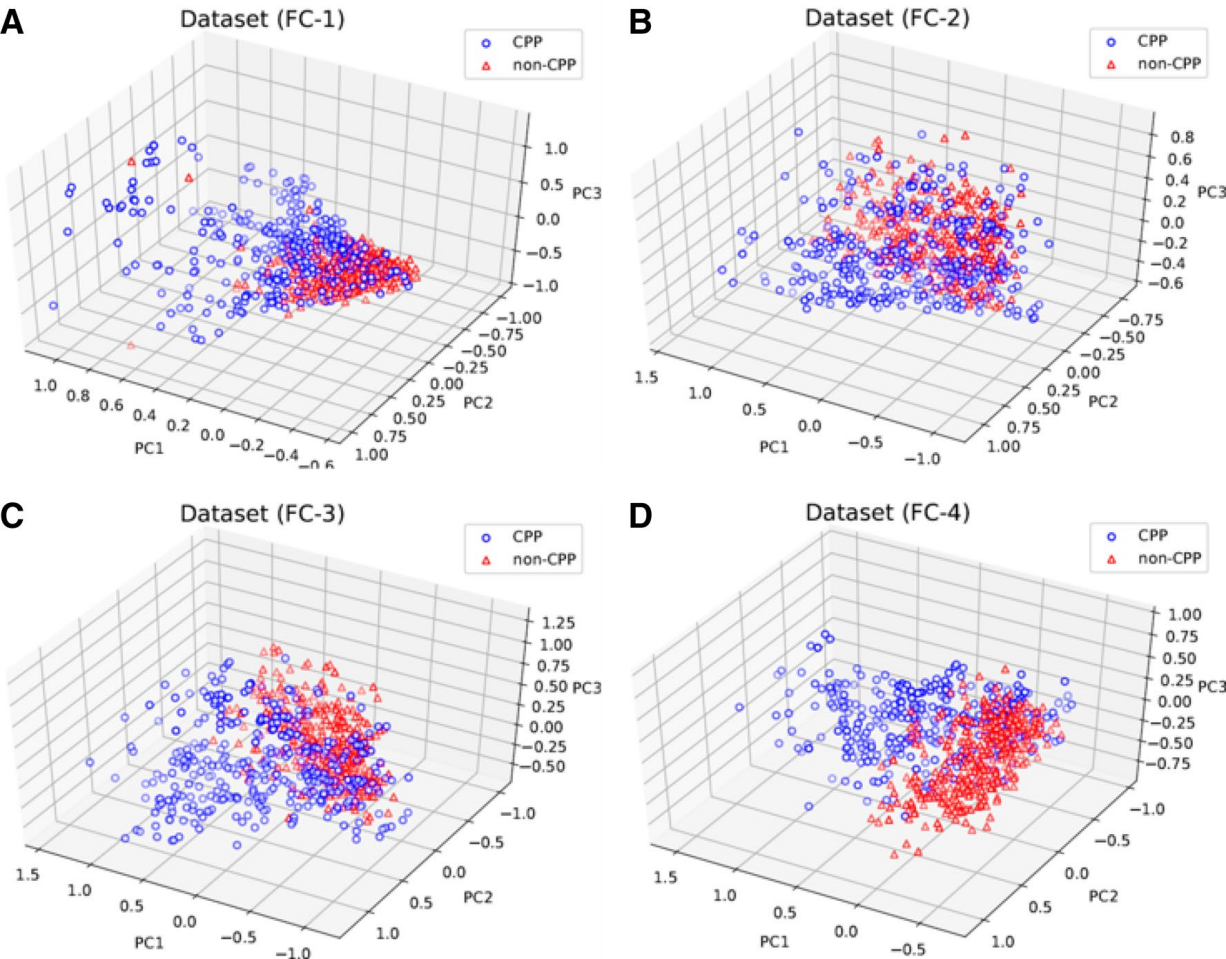
Analysis of 3D dimensionality reduction using PCA of the sequence- and structure-based descriptors present in FC-1 to FC-4. Panel (**A**) 3D PCA of FC-1 showing a contribution of explained variance ratio of 10.93% (PC1), 7.26% (PC2), and 6% (PC3), and cumulative explained variance ratio (CEVR) of 24.19%. (**B**) 3D PCA of FC-2 showing a contribution of explained variance ratio of 48.9% (PC1), 21.94% (PC2), and 14.34%(PC3), and CEVR = 85.19%. (**C**) 3D PCA of FC-3 showing a contribution of explained variance ratio of 16.31% (PC1), 12.03% (PC2), and 7.22% (PC3) and CEVR = 35.58%. (**D**) 3D PCA of FC-4 showing a contribution of explained variance ratio of 17.81% (PC1), 12.48% (PC2), and 8.93% (PC3), and CEVR = 39.29%.

The 3D PCA analyses of all datasets showed that FC-1 (Fig. 6A) and FC-2 (Fig. 6B) did not provide a clear differentiation between the CPPs and non-CPPs, which can be verified with the high level of overlap in the two groups of peptides. The normalized Bhattacharyya coefficient (BC) obtained values for FC1 equal to 0.361 (PC1), 0.234 (PC2), and 0.130 (PC3) and for FC2 values equal to 0.033 (PC1), 0.374 (PC2), and 0.045 (PC3).

In contrast, FC-3 (Fig. 6C) and FC-4 (Fig. 6D) reached lower overlap in the PCs, obtaining BC values equal to 0.342 (PC1), 0.061 (PC2), and 0.034 (PC3), thus indicating that these two FCs have more separability between CPPs and non-CPPs classes.

The Kruskal–Wallis H test applied among the three principal components of each 3D PCA also showed that there is no significant difference between FC-3 and FC-4, where the statistical hypotheses comparing the distribution of samples in PC1, PC2, and PC3 achieved *p* value of 0.826, 0.920, and 0.101, respectively, which indicates that the three PCs have similar distributions. These results confirmed that the optimized composition of structure- and sequence-based descriptors (FC-4) provided more significant information when compared with the other FCs, which directly impacted their cell membrane permeability prediction.

In contrast to previous ML-based approaches^31,34^, our findings demonstrated that the combination of sequence- and structure-based descriptors related to molecule bioavailability improved the prediction of CPPs’ structures. Structural factors, such as the presence of cyclic chains^92,93^, the secondary structure composition^94^, as well as, the shape, structure complexity, and 3D-pattern of constituting atoms^95^ have been shown to have a considerable influence on membrane penetration. Our analyses demonstrated that the membrane penetration of CPPs is better predicted using hybrid features composition containing structural and physicochemical properties, as well as, information from the primary structure.

## Conclusions

We demonstrated that the proposed BChemRF-CPPred, with FC composed of an optimized combination of sequence-, and structure-based properties, has superior accuracy compared to FCs composed of only sequence- or only structure-based descriptors. The accuracy achieved by the proposed framework, using PDB input and sequence- and structural-based features (FC-4), was 90.66% in the independent test with natural and non-natural peptides, while in the test with only natural peptides, the models based on FASTA input, which used only sequence-based descriptors (FC-1), and based on PDB input, which used (FC-4), achieved accuracy values of 86.5% and 89.6%, respectively. These performances were better than the reached by some other ML-based tools that applied as input data only the sequence-based properties of the peptides. However, the framework based on PDB input and FC-4 achieved better performance than the model based on FASTA input and FC-1 in the prediction of natural peptides as CPPs in the independent test. These results not only proved that our tool has a greater ability to correctly predict CPPs, as employing the optimized combination of the analyzed properties has more significant information for the ML-based algorithms applied to the CPP prediction problem than sequence- or structural-based descriptors analyzed separately. Finally, in addition to the Trojan metaphor applied for CPPs in drug delivery, in the present study, we demonstrated that these peptides, due to a highly diverse mechanism of membrane permeation that includes pore formation and endocytosis, also break some well-established chemical rules applied to predict the bioavailability of drugs. Similarly, the mythical Trojan horse broke the war rules.

## Material and methods

### Datasets of CPPs and non-CPPs structures

Our datasets of peptide structures were obtained from two curated and validated CPP databases. The CPP structures were obtained from CPPsite2.0, a chemo-structural database with more than 1700 validated experimental CPPs with different structural properties (linear/cyclic; and modified/non-natural residues) and a wide range of application for cargo transportations into the cell^96^. Moreover, 411 CPPs and 411 non-CPPs were obtained from the C2Pred server^35^. Additionally, we also obtained 112 CPP and 37 non-CPP structures from previous published works and pharmaceutical catalogs^32,97,98^.

The BCheRF-CPPred algorithm was trained and tested with datasets composed of primary and tertiary structure of peptides in FASTA (only natural peptides) and PDB (natural and synthetic peptides) formats, respectively. Peptides without resolved structures in PDB were predicted using the PEP-FOLD3 server^99^, and the peptides’ features were extracted to compose the CPP and non-CPP datasets.

The PEP-FOLD has been reported with high accuracy in the prediction of peptide structures obtaining the lowest energy conformations differing by 3.3 Å of RMSD-Ca from the experimental structures^99^. In addition, it is important to highlight that the structure-based descriptors (NRT, NAR, cLogP, HBA, HBD, etc.) analyzed in the present study are not related to the peptide folding, i.e., formation of secondary (α-helices and β-strands) and tertiary structures.

In the pre-processing stage, the general dataset was filtered regarding peptide length, which was limited to between 5 and 30 amino acid residues, and the duplicates and outliers (z-score ≥ 3 in peptide features) structures were removed using the Python data analysis library (Pandas) for Python language^100^. Finally, we organized a training dataset with 300 CPPs and 300 non-CPPs and an independent test dataset with 75 CPPs and 75 non-CPPs (Tables S5 and S6). Both datasets were balanced with a random selection of the structures.

### Calculation of sequence- and structure-based descriptors

The molecular properties related to cell membrane permeation were calculated for CPPs and non-CPPs libraries using both PDB and FASTA format.

We selected the following twelve structure-based descriptors: molecular weight (MW), number of rotatable bonds (NRB), topological polar surface area (tPSA), fraction of sp3-hybridized carbon atoms (Fsp^3^) (Eq. 1), 1-octanol/water partition coefficient (cLogP), number of aromatic rings (NAR), number of hydrogen bond donors (HBD), and number of hydrogen bond acceptors (HBA), number of primary amino groups (NPA), number of guanidinium groups (NG), net charge (NetC), and number of negatively charged amino acids (NNCAA) at pH = 7.4.

We also selected two amino acid composition AAC descriptors: fraction of arginine residues (f[Arg], Eq. 2) and lysine (f[Lys], Eq. 3). We also used two categories of sequence-based descriptors. The first one refers to 40 dipeptide composition (DPC, Eq. 4) selected from the best Kendall’s correlation values (dipeptides: RR, KK, KR, RQ, RK, WR, WK, NR, KW, WF, RS, FQ, RW, RI, QR, GR, RM, IW, RL, QN, ET, CN, PG, PL, GI, TV, FC, FG, GP, LS, SE, CV, GT, FL, CC, VC, GA, LG, GF, and GL).

The second one refers to 22 descriptors of the pseudo-amino acid composition (PseAAC)^88^, which are related to the hydrophobicity (${H}_{1}$), hydrophilicity (${H}_{2}$), and side-chain mass ($M$) along with the local sequence order, and can be calculated according to Eqs. (5) and (6), where *L* is the total residues content in peptide, λ is the correlation factor that reflects the sequence order of all the most contiguous residues along a protein chain, and ${R}_{i}$ is the *i*th amino acid. These properties were selected based on the general composition of CPP sequences^14^.

1 $${\mathrm{Fsp}}^{3}= \frac{{\mathrm{number}}\,{\mathrm{of}}\,{\mathrm{sp}}^{3}\,{\mathrm{hybridized}}\,{\mathrm{carbons}}}{{\mathrm{total}}\,{\mathrm{carbon}}\,{\mathrm{count}}}$$

2 $$\mathrm{f}[\mathrm{Arg}]= \frac{{\mathrm{number}}\,{\mathrm{of}}\,{\mathrm{arginine}}\,{\mathrm{residues}}}{{\mathrm{total}}\,{\mathrm{residues}}\,{\mathrm{count}}}$$

3 $$\mathrm{f}[\mathrm{Lys}]= \frac{{\mathrm{number}}\,{\mathrm{of}}\,{\mathrm{lysine}}\,{\mathrm{residues}}}{{\mathrm{total}}\,{\mathrm{residues}}\,{\mathrm{count}}}$$

4 $${\text{DPC}}_{j}= \frac{{\mathrm{number}}\,{\mathrm{of}}\,{\mathrm{dipeptides}}(j)}{{\mathrm{total}}\,{\mathrm{number}}\,{\mathrm{of}}\,{\mathrm{all}}\,{\mathrm{possible}}\,{\mathrm{dipeptides}}}$$

5 $$ PseAAC_{j} = \frac{1}{L - j}\mathop \sum \limits_{i = 1}^{L - j} \theta \left( {R_{i} ,R_{i + j} } \right), \quad 1 \le j \le 20 + \lambda \;\;\; {\text{and}}\;\;\;\lambda = 2 $$

6 $$ \theta \left( {R_{i} ,R_{i + j} } \right) = \frac{1}{3}\left\{ {\left[ {H_{1} \left( {R_{i} } \right) - H_{1} \left( {R_{i + j} } \right)} \right]^{2} + \left[ {H_{2} \left( {R_{i} } \right) - H_{2} \left( {R_{i + j} } \right)} \right]^{2} + \left[ {M\left( {R_{i} } \right) - M\left( {R_{i + j} } \right)} \right]^{2} } \right\} $$

Table 4 shows how all the descriptors were grouped into four different feature compositions, named FC-1 to FC-4. FC-1 grouped only amino acid composition and sequence-based descriptors, FC-2 used the twelve structure-based properties, FC-3 is the grouping of all analyzed descriptors, and the FC-4 grouped the most well-correlated sequence- and structure-based descriptors, according to Kendall’s correlation.

**Table 4:** Distribution of structure-, and sequence-based descriptors in four feature compositions used in BChemRF-CPPred.

Feature composition	Structural	AAC	DPC	PseAAC
**Molecular descriptors**				
FC-1	–	f[Lys], f[Arg]	40 DPCs*	22 PseAACs
FC-2	MW, cLogP, tPSA, Fsp^3^, NRB, HBD, HBA, NAR, NPA, NG, NetC, NNCAA	–	–	–
FC-3	MW, cLogP, tPSA, Fsp^3^, NRB, HBD, HBA, NAR, NPA, NG, NetC, NNCAA	f[Lys], f[Arg]	40 DPCs*	22 PseAACs
FC-4	MW, cLogP, Fsp^3^, HBA, NAR, NPA, NG, NetC, NNCAA	f[Lys], f[Arg]	10 DPCs^#^	22 PseAACs

*The 40 DPCs descriptors previously cited in this session.

^#^The 10 DPCs descriptors: RR, KK, KR, RQ, RK, GL, GF, LG, GA, VC.

The sequence- and structure-based descriptors were calculated by the RDKit^101^ package that uses Python language, except for the DPC and PseAAC that were calculated using PyBioMed^102^ package, and the NetC that was extracted from structures using Biopython package^103^.

To calculate some structure-based descriptors from PDB or FASTA format, the RDKit constructs a molecular structure of a peptide reading the file information. For PBD format, the package read the atoms, the sequence number, and the coordinates present in the file to form a graph with atomic bonds and dihedral angles that represents the molecule as a computational object since the vertice of the graph is an atom and the edge is the bond. To construct the 3D representation of the peptide using FASTA format, the RDKit reads the primary structure of the peptide and implements the graph theory using a list of predefined structures that matches with the conformation of the residues and their neighboring. This information can be consulted in RDKit API documentation in www.rdkit.org/docs/cppapi/ROMol_8h_source.html.

### Framework structure

**Figure 7:**
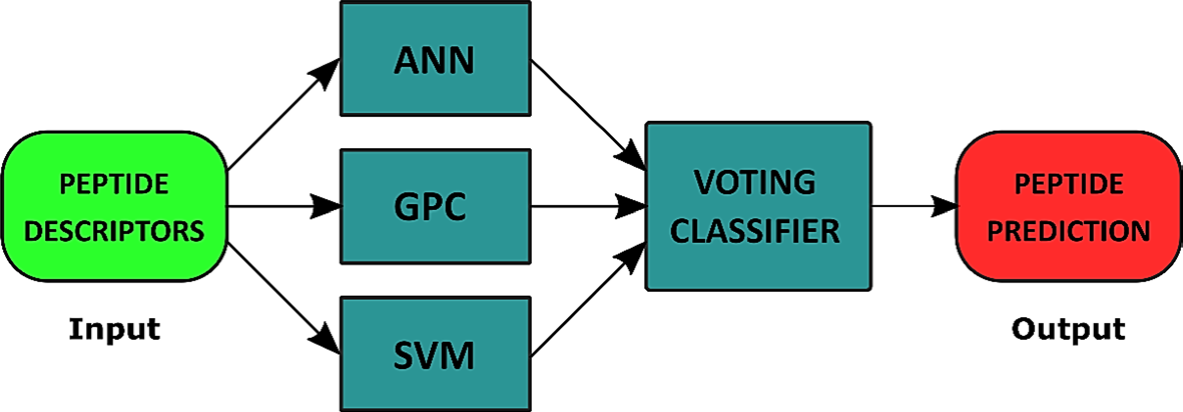
General structure of BChemRF-CPPred framework with ANN, GPC and SVM machine learning algorithms.

An ANN, MLP architecture^104^, GPC^105^, and SVM^106^ were employed in the BChemRF-CPPred to predict CPP permeability. Each ML-based algorithm received structure- and sequence-based descriptors to predict CPP and non-CPP structures using a probability scale that ranges from 0 to 1, where values > 0.5 were applied for CPPs and values ≤ 0.5 were applied for non-CPPs. The voting classifier calculates the average among the estimated probabilities, and the result provides a prediction of CPPs using binary labels, where 0 corresponds to non-CPPs and 1 to CPPs (Fig. 7).

**Figure 8:**
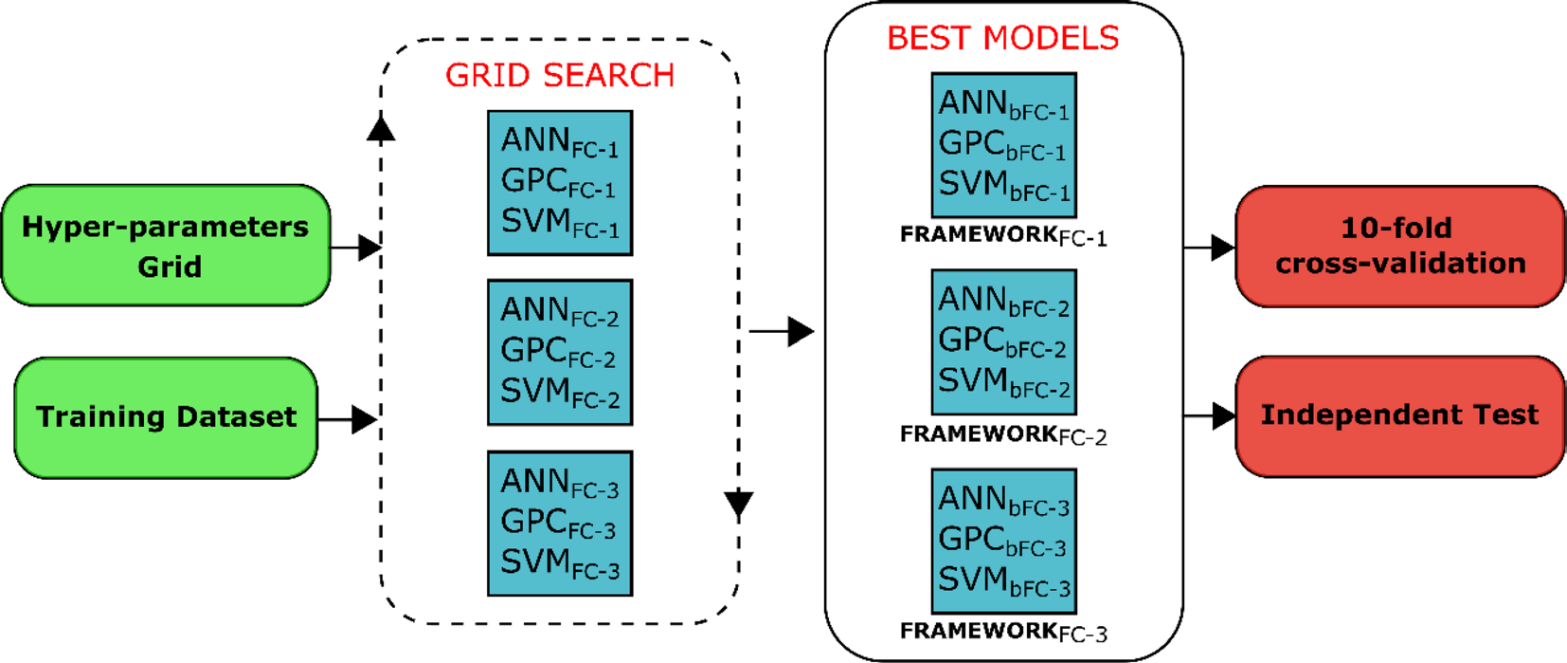
Process of hyper-parameters tuning applied for ANN, GPC, and SVM by FC using Grid Search method. The best models obtained in *x*-th feature composition (ANN_bFC-X_, GPC_bFC-X_, SVM_bFC-X_) were used to compose the respective framework.

The MLs’ hyper-parameters were tuned using Grid Search, a method applied for optimization of parameters using cross-validation over exhaustive search in a parameter grid. This method was applied to each algorithm by FC to obtain the best classifier model for the tenfold cross-validation and independent tests (Fig. 8). The range of the searching parameters adjusted for each ML-based algorithm and their best model are shown in Tables S3 and S4, respectively. All frameworks and their configuration processes were implemented using the Scikit-learn package for Python language.

### Calculation of information gain

The process of data mining to explore the information gain provided by each FC in the peptide dataset was based on extremely randomized trees^107^ and principal component analysis^106,108^ algorithms.

Extremely randomized trees are ensembles of unpruned decision trees algorithms that splits nodes by randomly-generated cut-points. This technique computes the importance of features using information entropy criterion. The higher is the entropy, the higher is the amount of information provided by the data.

Principal component analysis is an unsupervised machine learning technique used to reduce a high-dimensional dataset in a smaller dimensional representation, which is called principal components (PC). This algorithm turns out to be more feasible for the understanding of sample distribution in space.

ERT and PCA were implemented using Scikit-learn package and applied in the CPP structure library containing the peptides from training and independent test datasets.

### Web-server development

We developed a user-friendly web-server to implement the BChemRF-CPPred, which was coded using Flask, HTML, CSS, and JavaScript programming languages. The web server is freely available for academic use at http://comptools.linc.ufpa.br/BChemRF-CPPred.

In the “Prediction” session the user can select the primary structure (FASTA format) or the tertiary structure (PDB format) as input of peptides in BChemRF-CPPred, then the user can upload the desirable files and selects the intended feature composition (FC-1, FC-2, FC-3, or FC-4) to perform the prediction. In the “Download” button the user can download examples of CPPs and non-CPPs structures to test the server prediction. The “How to Use” button provides a brief explanation of the framework and how to use the web-server.

## Supplementary Information

Supplementary Informations.
