# Supplementary material for: Predicting cell-penetrating peptides using machine learning algorithms and navigating in their chemical space
Source: Sci Rep. 2021 Apr 7;11:7628. doi: 10.1038/s41598-021-87134-w (PMC8027643; doi:10.1038/s41598-021-87134-w)
Supplement: Supplementary file 1 — Supplementary Informations. [file 41598_2021_87134_MOESM1_ESM.docx]

**Predicting cell-penetrating peptides using machine learning algorithms and navigating in their chemical space**

Ewerton Cristhian Lima de Oliveira^1^, Kauê Santana^2,*^, Luiz Josino^3^, Anderson Henrique Lima e Lima^3,*^ and Claudomiro de Sales Júnior^1,*^

*^1^Institute of Technology. Federal University of Pará, 66075-110, Belém, Pará, Brazil*

*^2^Institute of Biodiversity. Federal University of Western Pará,* 68040-255, *Santarém, Pará, Brazil.*

*^3^Laboratório de Planejamento e Desenvolvimento de Fármacos, Instituto de Ciências Exatas e Naturais, Universidade Federal do Pará, 66075-110, Belém, Pará, Brasil.*

*Authors for correspondence:

1. Anderson Henrique Lima e Lima. Laboratório de Planejamento e Desenvolvimento de Fármacos, Instituto de Ciências Exatas e Naturais, Universidade Federal do Pará, 66075-110, Belém, Pará, Brasil. E-mail: anderson@ufpa.br

2. Kauê Santana. ^2^Institute of Biodiversity. Federal University of Western Pará, 68040-255, Vera Paz street, s/n Salé. Santarém Pará Brasil. Phone number +55 93 2101-6771. E-mail: [kaue.costa@ufopa.edu.br](mailto:kaue.costa@ufopa.edu.br).

3. Claudomiro de Souza de Sales Júnior: Institute of Technology. Federal University of Pará, 66075-110, Belém, Pará, Brazil. E-mail: [claudomiro.sales@gmail.com](mailto:claudomiro.sales@gmail.com).

ORCID of the authors:

Ewerton Cristhian Lima de Oliveira: 0000-0002-2338-7178.

Kauê Santana: 0000-0002-2735-8016.

Luiz Josino: 0000-0001-7482-9303.

Anderson Henrique Lima e Lima: 0000-0002-8451-9912.

Claudomiro de Sales Júnior: 0000-0002-2735-1383


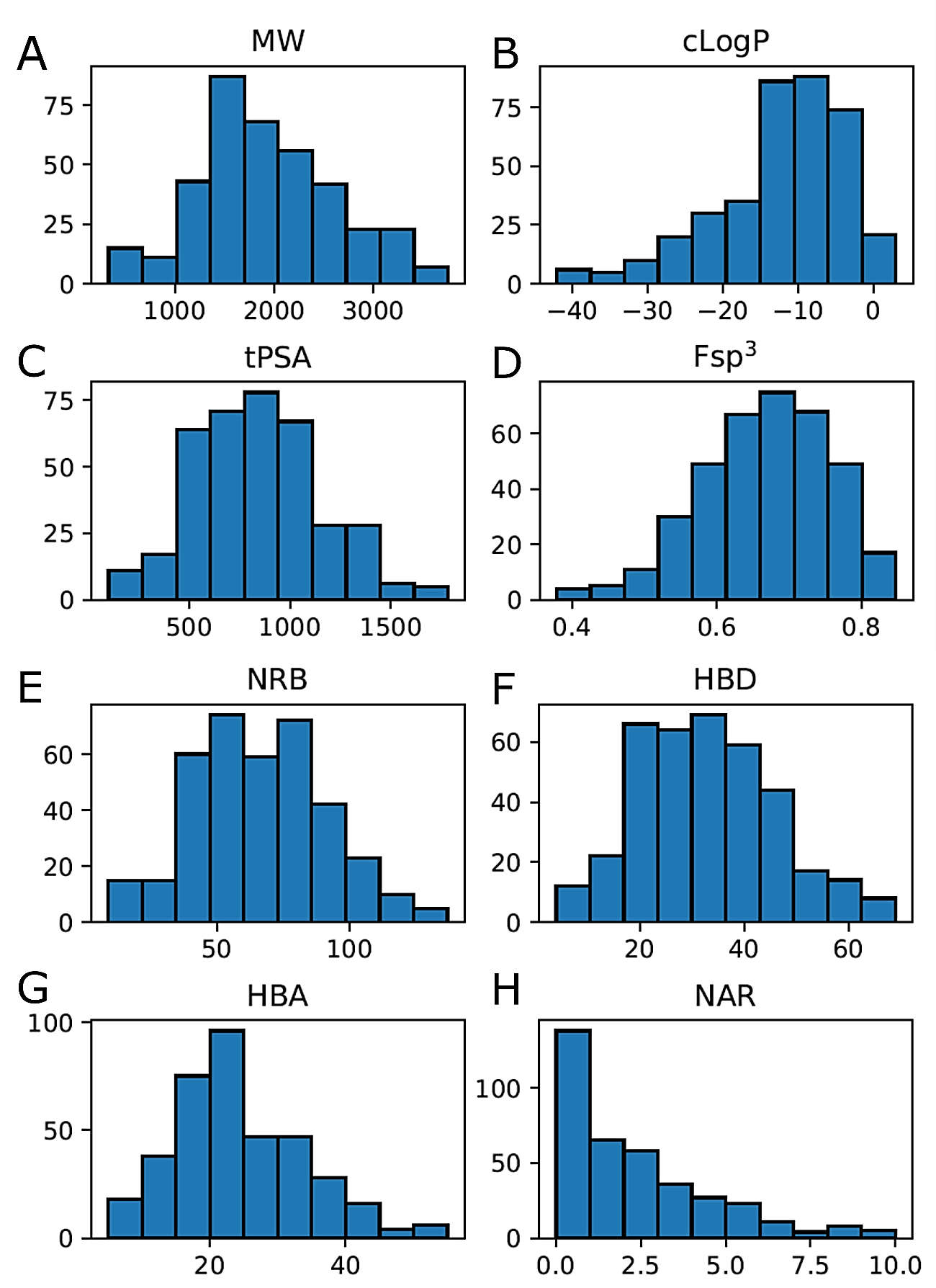


Figure S1. Histograms showing the distribution of the structure-based properties of the analyzed CPPs structures. (**A**) Molecular weight. (**B**) 1-octanol/water partition coefficient. (**C**) Topological polar surface area. (**D**) Fraction of sp^3^-hybridized carbon atoms. (**E**) Number of rotatable bonds. (**F**) Hydrogen bond donors. (**G**) Hydrogen bond acceptors. (**H**) Number of aromatic rings.


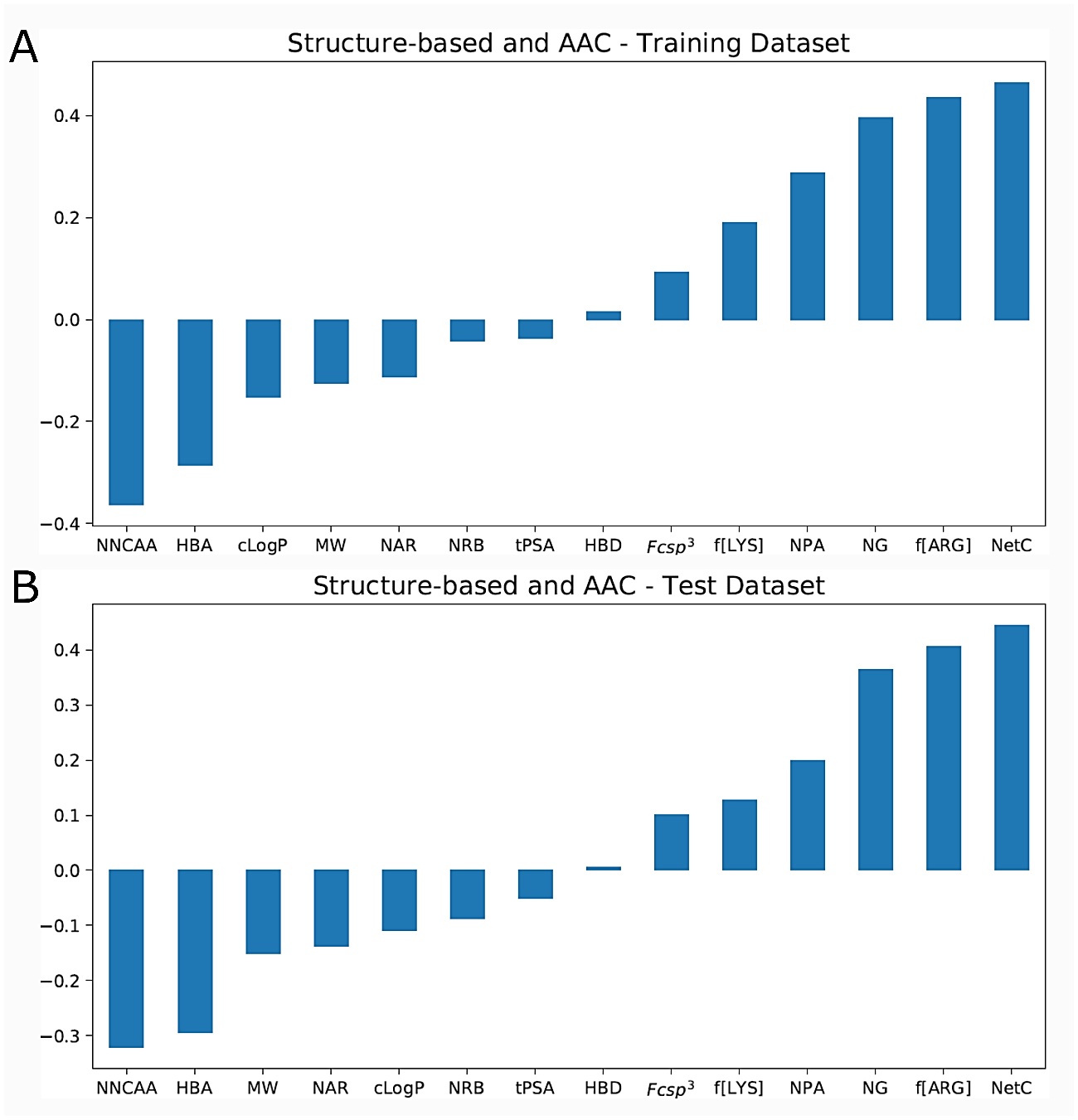


Figure S2. Kendall’s correlation of the analyzed sequence- and structure-based demonstrating the relevance of these properties to CPPs’ prediction. (**A**) Training dataset; (**B**) Independent test dataset.


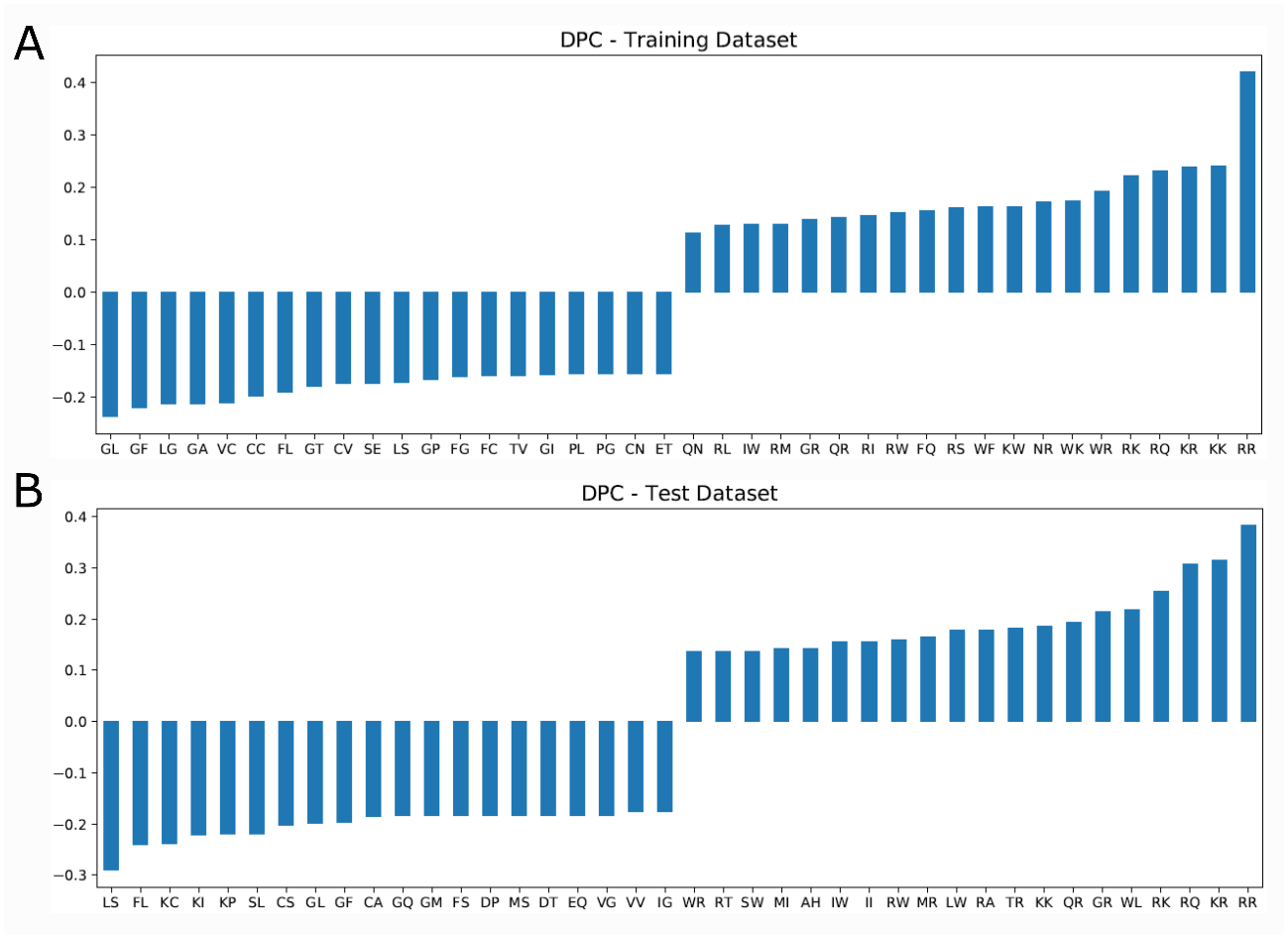


Figure S3. Kendall’s correlation of the analyzed dipeptide composition (DPC) descriptors demonstrating the relevance of these properties to CPPs’ prediction. (**A**) Training dataset; (**B**) Independent test dataset.


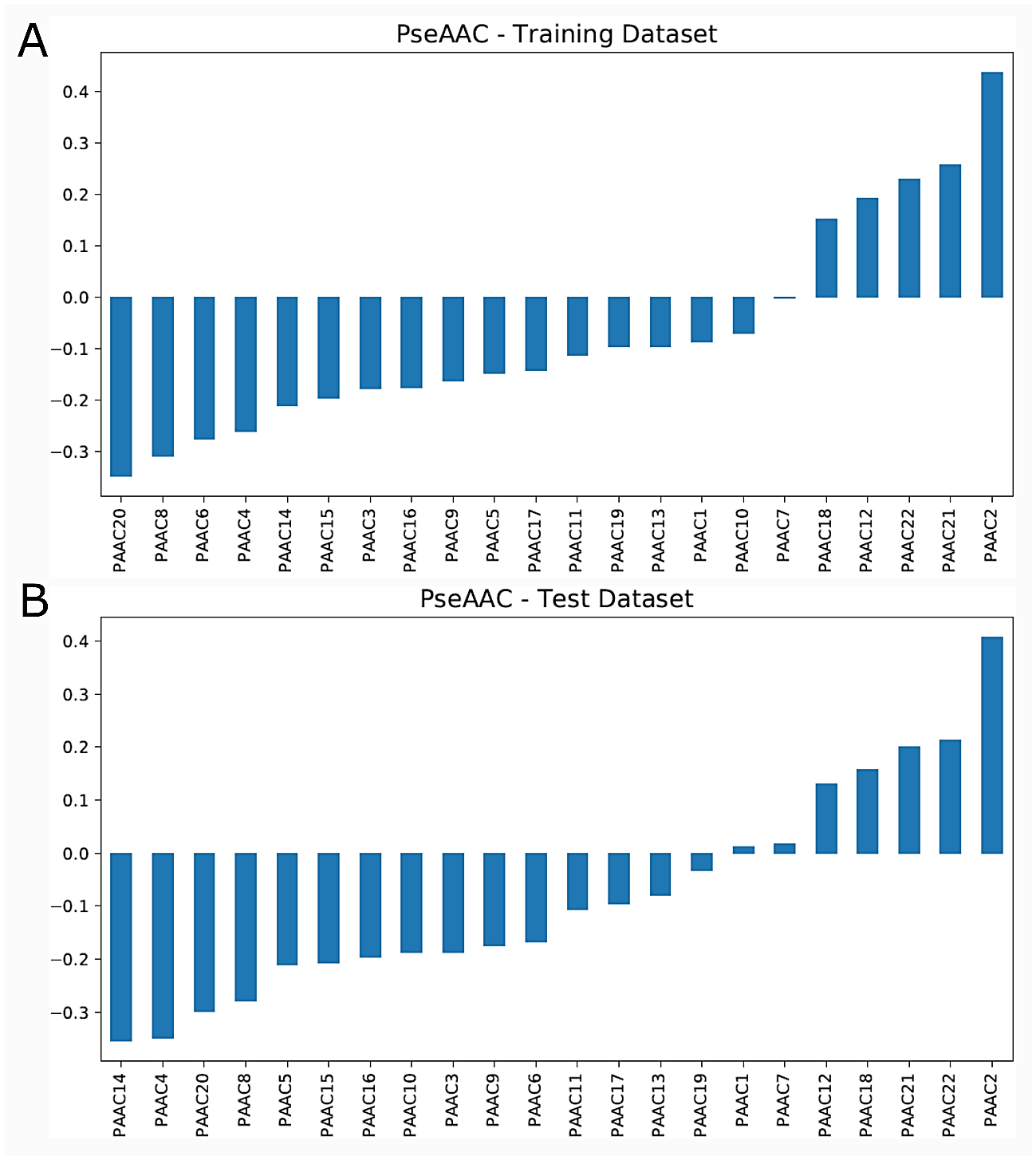


Figure S4. Kendall’s correlation of the analyzed pseudo amino acid composition (PseAAC) descriptors demonstrating the relevance of these properties to CPPs’ prediction. (**A**) Training dataset; (**B**) Independent test dataset.

| **Molecular properties** | **Oral drugs** | | **Peptides** | | |
| --- | --- | --- | --- | --- | --- |
|  | **Lipinski (2001) and Veber (2002)** | **Doak et al. (2014)** | **Santos et al. (2016)^*^** | **Diaz-Eufracio et al.**  **(2018)^**^** | **Our study^#^** |
| **MW** | $\leq$ 500 | $\leq$ 1,000 | $\leq$ 700 | 27.03 $\leq$MW $\leq$5,036.65 | 331.48 $\leq$ MW$\leq$ 3,750.51 |
| **cLogP** | $\leq$5 | -2 $\leq$ cLogP$\leq$10 | $\leq$7.5 | -17.87 $\leq$ cLogP$\leq$39.89 | -42.12 $\leq$ cLogP $\leq$ 2.97 |
| **tPSA** | $\leq14$0 | $\leq2$50 | $\leq$200 | $\leq$ 2,064.83 | 101.29 $\leq$ tPSA $\leq$ 1,782.83 |
| **Fsp^3^** | - | - | $\leq$0.55 | - | 0.37 $\leq$ Fsp^3^$\leq$ 0.84 |
| **NRB** | $\leq$ 10 | $\leq20$ | $\leq$ 20 | $\leq$209 | 9$\leq$ NRB $\leq$137 |
| **HBD** | $\leq$ 5 | $\leq6$ | $\leq$ 5 | $\leq$76 | 4 $\leq$ HBD $\leq$ 69 |
| **HBA** | $\leq1$0 | $\leq15$ | $\leq$10 | $\leq$71 | 5 $\leq$ HBA $\leq$55 |
| **NAR** | - | - | - | - | $\leq$ 10 |

Notes:

* Investigated oral available peptides; ** Investigated the linear and cyclic pentapeptides; # values obtained in the two peptides libraries (training and test datasets of CPPs).

Table S1. Comparison between previous chemical spaces identified for peptides and commercially available drugs.

| **Descriptors** | **80^th^ Percentile** | **90^th^ Percentile** | **95^th^ Percentile** | **Max – Min Limits** |
| --- | --- | --- | --- | --- |
| MW (Da) | 2,521.46 | 2,877.66 | 3,129.09 | 331.48 – 3,750.51 |
| cLogP | -4.971 | -2.61 | -1.285 | -42.120 - 2.976 |
| tPSA (Å^2^) | 1,090.97 | 1,282.15 | 1,381.66 | 101.29 – 1,782.83 |
| Fsp^3^ | 0.746 | 0.784 | 0.800 | 0.378 - 0.847 |
| NRB | 87 | 98.60 | 110 | 9 – 137 |
| HBD | 44.00 | 50.00 | 56.30 | 4 – 69 |
| HBA | 32.00 | 37.60 | 42 | 5 – 55 |
| NAR | 4 | 5 | 6 | 0 – 10 |
| NetC | 6.66 | 7.55 | 8.54 | -1.74 – 13.53 |
| NNCAA | 2 | 2 | 4 | 0 - 6 |
| NPA | 11 | 14 | 16 | 0 - 24 |
| NG | 6 | 6 | 8 | 0 - 10 |
| f[Arg] | 0.375 | 0.500 | 0.578 | 0 - 0.875 |
| f[Lys] | 0.222 | 0.285 | 0.367 | 0 - 0.533 |

Table S2: Calculated 80^th^, 90^th^, and 95^th^ percentiles of the analyzed CPP structures from training and test datasets using some structure-based and sequence-based descriptors.

| **ANN** | | **GPC** | | **SVM** | |
| --- | --- | --- | --- | --- | --- |
| Activation  Function | FC-1: Relu  FC-2: Relu  FC-3: Relu  FC-4: Relu | Kernel | FC-1: Sigmoid  FC-2: RBF  FC-3: RBF  FC-4: RBF | Kernel | RBF |
| Number of Hidden Layers | FC-1: 2  FC-2: 2  FC-3: 1  FC-4: 2 | α | FC-1: 1  FC-2: 1  FC-3: 1  FC-4: 1 | *C* | FC-1: 500  FC-2: 1000  FC-3: 2000  FC-4: 5000 |
| Number of Neurons by Layer | FC-1: 70  FC-2: 70  FC-3: 60  FC-4: 80 | Length Scale (RBF) | FC-1: 0.05  FC-2: 0.01  FC-3: 0.05  FC-3: 0.05 | γ | FC-1: 0.005  FC-2: 0.1  FC-3: 0.01  FC-4: 0.005 |

Table S3: Hyperparameters of the best models of ANN, GPC, and SVM obtained for the FC achieved by Grid Search.

| **ANN** | | **GPC** | | **SVM** | |
| --- | --- | --- | --- | --- | --- |
| Activation  function | [Relu; Logistic] | Kernel | [α*RBF; α*Rational Quadratic] | Kernel | RBF |
| Number of hidden layers | [1; 2] | α | [1; 2] | *C* | [500; 1000; 2000; 5000] |
| Number of neurons by Layer | [30; 40; 50; 60; 70; 80] | Length scale (RBF) | [0.01; 0.02; 0.05] | γ | [0.005; 0.01; 0.1; 0.2] |
| Number of iterations* | 1500 | Length scale (RQ) | [0.01; 0.05] | Number of iterations* | 150000 |
| Training  algorithm* | Adam | Number of iterations* | 100 | - | - |
| - | - | Training algorithm* | L-BFGS-B | - | - |

* Prefixed parameters.

Table S4: Parameters used in the Grid Search for tuning ANN, GPC, and SVM algorithms.

| **Sequence (CPP)** | **Reference** | **Origin** | **Model** | **Sequence (non-CPP)** | **Reference** | **Origin** | **Model** |
| --- | --- | --- | --- | --- | --- | --- | --- |
| KETWWETWWTEWSQPKKRKV | pep-1 | DB1 | COMP | TERQIKIWFQNRRMK | pAntp41-55 | DB1 | COMP |
| TRSSRAGLQFPVGRVHRLLRK | Buforin | DB1 | COMP | AHALCLTERQIKSNRRMKWKKEN | pAntpHD 48S | DB1 | COMP |
| TAKTRYKARRAELIAERR | Phi21 N (12-29) | DB1 | COMP | FITKALGISYGRKKRRQC | ptat7 | DB1 | COMP |
| KFHTFPQTAIGVGAP | hCT18-32 | DB1 | COMP | ILRRRIRKQAHAHSK | pVEC(4-18) | DB1 | COMP |
| TRQARRNRRRWRERQR | HIV-1 rev | DB1 | COMP | AGCKNFFWKTFTSC | Somatostatin 14 | DB1 | COMP |
| ALWKTLLKKVLKA | K4-S4(1-13)a | DB1 | COMP | GWTLNSAGYLLGPHAI | Galanin (1-16) | DB1 | COMP |
| LLIILRRRIRKQAHAHSK | pVEC | DB1 | COMP | DFDMLRCMLGRVYRPCWQV | HCM | DB1 | COMP |
| KLALKALKALKAALKLA | MAP | DB1 | COMP | WSYGLRPG | [1] | DB1 | COMP |
| AAVALLPAVLLALLAKNNLKECGLY | [1] | DB1 | COMP | KKKQYTSIHHGVVEVD | [1] | DB1 | COMP |
| KMTRAQRRAAARRNRWTAR | [1] | DB1 | COMP | GWTNLSAGYLLGPPPGFSPFR | [1] | DB1 | COMP |
| LLIILRRPIRKQAHAHSK | [1] | DB1 | COMP | PVVHLTLRQAGDDFSR | [1] | DB1 | COMP |
| LLIILRRRIRKQAHAHSA | [1] | DB1 | COMP | EILLPNNYNAYESYKYPGMFIALSK | [1] | DB1 | COMP |
| SWLGRQLRIAGKRLEGRSK | [1] | DB1 | COMP | QNLGNQWAVGHLM | [1] | DB1 | COMP |
| GAARVTSWLGRQLRIAGKRLEGRSK | [1] | DB1 | COMP | VPLPAGGGTVLNQDVPARQPLGG | [3] | C2Pred | COMP |
| AAVALLPAVLLALLAPVQRKQKLMP | [1] | DB1 | COMP | AATAATPATAATPATAARA | [3] | C2Pred | COMP |
| RQGAARVTSWLGRQLRIAGKRLEGR | [1] | DB1 | COMP | IIGAIAAALPHVINAIKNTFG | [3] | C2Pred | COMP |
| GYGNCRXFKQKPRRD | [2] | CPPsite 2 | COMP (CPPSite ID: 1266) | PSCVCSGFETSGIHFC | [3] | C2Pred | COMP |
| IGCRX | [2] | CPPsite 2 | COMP (CPPSite ID: 1642) | SCIKHGDFCDGDNDDCQCCRDNGF | [3] | C2Pred | COMP |
| YGRKKRRQRRRGTALDWSWLQTE | [3] | C2Pred | COMP | YQLLQELCCQHL | [3] | C2Pred | COMP |
| GRKGKHKRKKLP | [3] | C2Pred | COMP | IVQQCTSGICSLYQENYCN | [3] | C2Pred | COMP |
| KFLNRFWHWLQLKPGQPMY | [3] | C2Pred | COMP | GIACGESCVFLGCFIPGCSCKSKVCYFN | [3] | C2Pred | COMP |
| RRRRRRRRRGPGVTWTPQAWFQWV | [3] | C2Pred | COMP | HGVSGHGQHGVHG | [3] | C2Pred | COMP |
| AEKVDPVKLNLTLSAAAEALTGLGDK | [3] | C2Pred | COMP | PVKVYPNGVQEETSEGFPLEF | [3] | C2Pred | COMP |
| WIIFKIAASXKK | [2] | CPPsite 2 | COMP (CPPSite ID: 1622) | NPRWEFRGKFVGVR | [3] | C2Pred | COMP |
| CXXRRRRXXC | [2] | CPPsite 2 | COMP (CPPSite ID: 2125) | LYISRQGFRPA | [3] | C2Pred | COMP |
| GLKKLARLFHKLLKLGC | [3] | C2Pred | COMP | GSSGMIPFPRV | [3] | C2Pred | COMP |
| VVLGKLYGRKKRRQRRR | [2] | CPPsite 2 | COMP | GWKSVFRKAKKVGKTVGGLALDHYLG | [3] | C2Pred | COMP |
| TSPLNIHNGQKL | [3] | C2Pred | COMP | LGQGSFRPSQQN | [3] | C2Pred | COMP |
| PSKRLLXNNLRR | [2] | CPPsite 2 | COMP (CPPSite ID: 1655) | WLSKTAKKLENSAKKRISEGIAIAIKGGSR | [3] | C2Pred | COMP |
| NYTTYKSHFQDR | [3] | C2Pred | COMP | GVLSNVIGYLKKLGTGALNAVLKQ | [3] | C2Pred | COMP |
| RKKRRQR | Tat (49-55) | CPPsite 2 | COMP (CPPSite ID: 1008) | SFHVFPPWMCKSLKKC | [3] | C2Pred | COMP |
| LIIFAIAASXKK | [2] | CPPsite 2 | COMP (CPPSite ID: 1631) | GLLSKVLGVGKKVLCGVSGLC | [3] | C2Pred | COMP |
| CGGKDCERRFSRSDQLKRXQRRXTGVKPFQ | b-WT1-pTj | CPPsite 2 | COMP (CPPSite ID: 2303) | CCSQDCLVCIPCCPN | [3] | C2Pred | COMP |
| MIIYRIAASHKK | [3] | C2Pred | COMP | QATVGDVNTDRPGLLDLK | [3] | C2Pred | COMP |
| RRQRRTSKLMKR | [3] | C2Pred | COMP | CGETCVTGTCYTPGCACDWPVCKRD | [3] | C2Pred | COMP |
| LILIGRRRRRRRRGC | LILIR8 (Alexa) | CPPsite 2 | COMP (CPPSite ID: 2691) | LKLKDILGKIKVILSHLNK | [3] | C2Pred | COMP |
| CRQIKIWFPNRRMKWKKC | Reduced linear penetratin | CPPsite 2 | COMP (CPPSite ID: 1131) | AFDHYGFTGGL | [3] | C2Pred | COMP |
| CRWRWKSSKK | Crot (27-39) derevative | CPPsite 2 | COMP (CPPSite ID: 1167) | CKSKGAKCSKLMYDCCSGSCSGTVGRC | [3] | C2Pred | COMP |
| SWAQHLSLPPVL | [3] | C2Pred | COMP | SENPSNSRNFIRL | [3] | C2Pred | COMP |
| GRQLRIAGRRLRRRSR | [3] | C2Pred | COMP | KPNPERFYAPM | [3] | C2Pred | COMP |
| LGTYTQDFNKFXTFPQTAIGVGAP | EGFP-hcT(9-32) | CPPsite 2 | COMP (CPPSite ID: 2226) | GSLTGLISMPRT | [3] | C2Pred | COMP |
| QWQRNMRKVR | M6 | CPPsite 2 | COMP (CPPSite ID: 1413) | PDERRQLNKIFLWDFCNSDSI | [3] | C2Pred | COMP |
| APWXLSSQYSRT | CTP | CPPsite 2 | COMP (CPPSite ID: 2588) | CCKVQCESCTPCC | [3] | C2Pred | COMP |
| GLLEALAELLEGLRKRLRKFRNKIKEK | [3] | C2Pred | COMP | DLWNSIKDMAAAAGRAALNAVTGMVNQ | [3] | C2Pred | COMP |
| AAVALLPAVLLALLAK | MPS | CPPsite 2 | COMP (CPPSite ID: 1791) | MPPSGLRLLPLLLPLPWLLVLTP | [3] | C2Pred | COMP |
| NYQWRCKNQN | ECP(32-41)R3Q | CPPsite 2 | COMP (CPPSite ID: 2024) | FLSFLLGPLIDLISKG | [3] | C2Pred | COMP |
| KFXTFPQTAIGVGAP | hCT(18-32) | CPPsite 2 | COMP (CPPSite ID: 1461) | LKKISQYYQKFAWPQYL | [3] | C2Pred | COMP |
| XRLRXALAXLLXKLKXLLXALAXRLRX | [2] | CPPsite 2 | COMP | ISCQDVKQSLAPCLPYVTGRAPKPA | [3] | C2Pred | COMP |
| KCRKKKRRQRRRKKPVVHLTLRQAGDDFSR | [3] | C2Pred | COMP | RGCREGGEFCGTLYEERCCSGWCFFVCV | [3] | C2Pred | COMP |
| AGYLLGXINLXXLAXLXXILC | TH peptide | CPPsite 2 | COMP (CPPSite ID: 2122) | GSSGLIPFGRT | [3] | C2Pred | COMP |
| KRIPNKKPGKKTTTKPTKKPTIKTTKK | [2] | CPPsite 2 | COMP | EEKMGFAKKCCAIGCSTEDFRMVC | [3] | C2Pred | COMP |
| ANIIXPLLXPIC | [2] | CPPsite 2 | COMP | RKYVMGHFRWDRFGRRNSSSSGSSGAGQKR | [3] | C2Pred | COMP |
| LNSAGYLLGKLKALAALAK | [2] | CPPsite 2 | COMP | SPANAQITRKRHKINSFVGLM | [3] | C2Pred | COMP |
| EEEAAKKK | [2] | CPPsite 2 | COMP | AKWKEDVIKLCSRELVRTQIAICG | [3] | C2Pred | COMP |
| RIKAERKRMRNRIAASKSRKRKLERIARGC | [3] | C2Pred | COMP | QYPLGQGSFRPS | [3] | C2Pred | COMP |
| VLGQSGYLMPMR | [3] | C2Pred | COMP | FLPFLAKILTGVL | [3] | C2Pred | COMP |
| RRKLSQQKEKK | [3] | C2Pred | COMP | KPSPDRFYGLM | [3] | C2Pred | COMP |
| GSRVQIRCRFRNSTR | [3] | C2Pred | COMP | SFPFFPPGICKRLKRC | [3] | C2Pred | COMP |
| YWLKLLKKWLKLWKKLLKLW | [2] | CPPsite 2 | COMP | YGGFLRRQFKVVTRSQEDPNAYSGELFDA | [3] | C2Pred | COMP |
| KLPCRSNTFLNIFRRKKPG | [3] | C2Pred | COMP | GIGKFLKKAKKGIGAVLKVLTTGL | [3] | C2Pred | COMP |
| CGRKKRRQRRAARPPQ | [2] | CPPsite 2 | COMP | HSEGTFSNDYSKYLETRRAQDFVQWLKNS | [3] | C2Pred | COMP |
| ACRGRGRGCGRGRGRCG | [2] | CPPsite 2 | COMP | AAEFPDFYDSEEQMGPHQEA | [3] | C2Pred | COMP |
| RLWMRWYSPTTRRAG | [2] | CPPsite 2 | COMP | FVSRHLCGSNLVETLYSVCQDDGFFYIPKD | [3] | C2Pred | COMP |
| GKKKRKLSNRESAKRSR | [3] | C2Pred | COMP | FLPMLAKLLSGFLGK | [3] | C2Pred | COMP |
| AAVALLPAVLLALLAPSGASGLDKRDYV | [3] | C2Pred | COMP | GKCGEINGSCDECYGGSVTCDCY | [3] | C2Pred | COMP |
| GWTLNSAGYLLGKINLKAPAALAKKIL | [2] | CPPsite 2 | COMP | HLPPPVHLPPPV | [3] | C2Pred | COMP |
| ACGRGRGRCRGRGRGCG | [2] | CPPsite 2 | COMP | GLVSSIGRALGGLLADVVKSKGQPA | [3] | C2Pred | COMP |
| LIIFRIAASXKK | [2] | CPPsite 2 | COMP | QPFPQPQQPFPQSQ | [3] | C2Pred | COMP |
| NYTTYKSXFQDR | [2] | CPPsite 2 | COMP | CCPGWELCCEWDDGW | [3] | C2Pred | COMP |
| RKKRRQRAR | [2] | CPPsite 2 | COMP | NCPYCVVYCCPPAYCEASGCRPP | [3] | C2Pred | COMP |
| LNSAGYLLGKALAALAKKIL | [2] | CPPsite 2 | COMP | DYDPRTEAPRRLPADDDEVDGEDRV | [3] | C2Pred | COMP |
| PPKKSAQCLRYKKPE | [3] | C2Pred | COMP | FLPKMSTKLRVPYRRGTKDYH | [3] | C2Pred | COMP |
| MIIYRDKKSX | [2] | CPPsite 2 | COMP | FLPLLAGVVANFLPQIICKIARKC | [3] | C2Pred | COMP |
| FFLIPKGRRRRRRRRGC | [3] | C2Pred | COMP | GLPVCGETCFGGRCNTPGCTCSYPICTRN | [3] | C2Pred | COMP |
| KXKLLXLLXLLALLWLXLLXLLKXK | [2] | CPPsite 2 | COMP | FFGHLFKLATKIIPSLFQ | [3] | C2Pred | COMP |
| VKRFKKFFRKLKKLV | [2] | CPPsite 2 | COMP | GFFGKMKEYFKKFGASFKRRFANLKKRL | [3] | C2Pred | COMP |
| RRRRRRRRRGD | [2] | CPPsite 2 | COMP | TCTLGTCYTAGCSCSWPVCTRNGVPICGE | [3] | C2Pred | COMP |
| KALKLKLALALLAKLKLA | [3] | C2Pred | COMP | GLFLDTLKGLAGKLLQGLKCIKAGCKP | [3] | C2Pred | COMP |
| ISFDELLDYYGESGS | [3] | C2Pred | COMP | ALCCYGYRFCCPNFR | [3] | C2Pred | COMP |
| RGDFK | [2] | CPPsite 2 | COMP | GLTPNMNSLFF | [3] | C2Pred | COMP |
| KLWMRWYSPWTRRYG | [2] | CPPsite 2 | COMP | FLGALIKGAIHGGRFIHGMIQNHHG | [3] | C2Pred | COMP |
| NAKTRRXERRRKLAIERGC | [2] | CPPsite 2 | COMP | FLPVLAGIAAKVVPALFCKITKKC | [3] | C2Pred | COMP |
| KKDGKKRKRSRKESYSVYVYKVLKQ | [3] | C2Pred | COMP | GRRKRKWLRRIGKGVKIIGGAALDHL | [3] | C2Pred | COMP |
| ACRRSRRGCGRRSRRCG | [2] | CPPsite 2 | COMP | NGVYCTKNKCTVDWAKATTCIAGMSIGGF | [3] | C2Pred | COMP |
| HIQLSPFSQSWR | [3] | C2Pred | COMP | GLFDIIKKVASVVGLASQ | [3] | C2Pred | COMP |
| MIIFKIAASXKK | [2] | CPPsite 2 | COMP | GHDFDQDDVNSSGEKDESLVRI | [3] | C2Pred | COMP |
| TARRITPKDVIDVRSVTTEINT | [2] | CPPsite 2 | COMP | TPVVNPPFLQQT | [3] | C2Pred | COMP |
| GLGSLLKKAGKKLKQPKSKRKV | [3] | C2Pred | COMP | LNLKALLAVAKKIL | [3] | C2Pred | COMP |
| TRQARRNRRRRWRERQR | Rev (34-50) | CPPsite 2 | EXPR (PDB code: 1RPV) | EPTWNNLKGMW | [3] | C2Pred | COMP |
| WEARLARALARALARXLARALARA | [2] | CPPsite 2 | COMP | AEPGADDAEEVEQKQLQ | [3] | C2Pred | COMP |
| ASMWERVKSIIKSSLAAASNI | [3] | C2Pred | COMP | GLGDILGLLGL | [3] | C2Pred | COMP |
| RIRMIQNLIKKT | [3] | C2Pred | COMP | IPPYCTIAPFGI | [3] | C2Pred | COMP |
| QIISRDLISX | [2] | CPPsite 2 | COMP | QQPFVQQQQPFVQQ | [3] | C2Pred | COMP |
| QAASRVENYMHR | [3] | C2Pred | COMP | GPYGGGGLVGALLG | [3] | C2Pred | COMP |
| GLKKLAELFXKLLKLG | [2] | CPPsite 2 | COMP | GCCPFPACTHTIICRCC | [3] | C2Pred | COMP |
| LNSAGYLLGKINLKALAALAKKIL | [2] | CPPsite 2 | COMP | MSPRPLAWALVL | [3] | C2Pred | COMP |
| GGAYVTRSSAVRLRSSVPGVRLLQ | [3] | C2Pred | COMP | EYDDMYTEKRPKVYAFGL | [3] | C2Pred | COMP |
| KWCFRVCYRGICYRRCRGK | [3] | C2Pred | COMP | GGCRIGPITWVCGG | [3] | C2Pred | COMP |
| RRIRPRP | [2] | CPPsite 2 | COMP | GLFTLIKCAYQLIAPTVACN | [3] | C2Pred | COMP |
| RRARRPRRLRPAPGR | [3] | C2Pred | COMP | FIGAVAGLLSKIF | [3] | C2Pred | COMP |
| RKKRRRESRKKRRRES | [2] | CPPsite 2 | COMP | ALWKTLLKGAGKVFGHVAKQFLGSQGQPES | [3] | C2Pred | COMP |
| YPRAARRAARR | [2] | CPPsite 2 | COMP | MAASPRNSVLLA | [3] | C2Pred | COMP |
| KKKEERADLIAYLKKA | [2] | CPPsite 2 | COMP | NPGTPQHLCGSHLVDALYLVCGPTGFFYNP | [3] | C2Pred | COMP |
| ACRGRGRGCRGRGRGCG | [2] | CPPsite 2 | COMP | DCCHNTQLPFIYKTCPEGCNL | [3] | C2Pred | COMP |
| RRRQRRKKR | [2] | CPPsite 2 | COMP | FIPLVSGLFSRLL | [3] | C2Pred | COMP |
| LIRLWSXLIXIWFQNRRLKWKKKGGC | [2] | CPPsite 2 | COMP | CAETCIYIPCFTEAVGCKCKDKVCYKN | [3] | C2Pred | COMP |
| KLALKAALKAWKAAAKLA | [2] | CPPsite 2 | COMP | KSDLLGALLSRNSPSSYGLPSRDMSTAY | [3] | C2Pred | COMP |
| NKPILVFY | [2] | CPPsite 2 | COMP | VYVPRYIANLY | [3] | C2Pred | COMP |
| RLXRRLXRRLXRLXR | [2] | CPPsite 2 | COMP | FKVQNQHGQVVKIFHH | [3] | C2Pred | COMP |
| RRLRXLRXXYRRRWXRFR | [2] | CPPsite 2 | COMP | GLVPNLLNNLGL | [3] | C2Pred | COMP |
| ERKKRRRE | [2] | CPPsite 2 | COMP | MTPPPLPARVDFSLAGALN | [3] | C2Pred | COMP |
| DRDRDRDRDR | [2] | CPPsite 2 | COMP | KIHPFAQTQSLVYP | [3] | C2Pred | COMP |
| KKLALXALXLLALLWLXLAXLALKK | [2] | CPPsite 2 | COMP | SLLADTQSGHRW | [3] | C2Pred | COMP |
| MAMPGEPRRANVMAHKLEPASLQLRSCA | [3] | C2Pred | COMP | GLWNKIKEAASKAAGKAALGFVNEMVG | [3] | C2Pred | COMP |
| ALIILRRRIRKQAXAXSK | [2] | CPPsite 2 | COMP | YIKVLRCRVVFQNEC | [3] | C2Pred | COMP |
| GSPWGLQXXPPRT | [2] | CPPsite 2 | COMP | FFPNVASVPGQVLLKKIFCAISKKC | [3] | C2Pred | COMP |
| LLYWFRRRXRXXRRRXRR | [2] | CPPsite 2 | COMP | GCIGRNESQKKDNVYKFKE | [3] | C2Pred | COMP |
| KKAAQIRSQVMTXLRVI | [2] | CPPsite 2 | COMP | MRKEFHNVLSSGQLLADKRPARDYNRK | [3] | C2Pred | COMP |
| GKYVSLTTPKNPTKRRITPKDV | [3] | C2Pred | COMP | FMGGLIKAATKIVPAAYCAITKKC | [3] | C2Pred | COMP |
| ARCSGSGSGCGSGSGSCGR | [2] | CPPsite 2 | COMP | VIVKAIATLSKKLL | [3] | C2Pred | COMP |
| CSSLDEPGRGGFSSESKV | [3] | C2Pred | COMP | ILGLLKGISALLS | [3] | C2Pred | COMP |
| ACSSSPSKXCGGGGRRRRRRRRR | [2] | CPPsite 2 | COMP | GPGSAICNMACRLEHGHLYPFCNCD | [3] | C2Pred | COMP |
| RRWFRRWRR | [2] | CPPsite 2 | COMP | GIVEQCCTSICSLYQLENYCN | [3] | C2Pred | COMP |
| RRVWRRYRRQRWCRR | [3] | C2Pred | COMP | AIEWEGIESGSVEQA | [3] | C2Pred | COMP |
| VKLPPP | [2] | CPPsite 2 | COMP | RCCKFPCPDSCRYLCC | [3] | C2Pred | COMP |
| NNNAAGRKRKKRT | [3] | C2Pred | COMP | FIGPLISALASLFKG | [3] | C2Pred | COMP |
| TPKTMTQTYDFS | [3] | C2Pred | COMP | MQKLQISVYIYLFMLIVAGPVDLNENSEQK | [3] | C2Pred | COMP |
| XRRRRRRR | [2] | CPPsite 2 | COMP | QGTTNIVCECCMKPCTLSELRQYCP | [3] | C2Pred | COMP |
| RILQQLLFIXFRIGCRXSRI | [2] | CPPsite 2 | COMP | SCIALTLVLVANSAPTTSSSTKETQQQLE | [3] | C2Pred | COMP |
| PRPRPLPFPRPG | [2] | CPPsite 2 | COMP | MHSSALLCCLVLLT | [3] | C2Pred | COMP |
| RQIKIWFQNARMKWKK | [2] | CPPsite 2 | COMP | FIGALLGPLLNLLK | [3] | C2Pred | COMP |
| GLKKLARLAXKLLKLGC | [2] | CPPsite 2 | COMP | SAGATANLPLRS | [3] | C2Pred | COMP |
| GKRRRRATAKYRSAH | [3] | C2Pred | COMP | VQQQQPLGQQQP | [3] | C2Pred | COMP |
| SRWRWKSSKK | [2] | CPPsite 2 | COMP | VVCNYRDVRFESIRLPGGPRGVNPVVSY | [3] | C2Pred | COMP |
| RKLTTIFPLNWKYRKALSLG | [3] | C2Pred | COMP | QGRLGTQWAVGHLM | [3] | C2Pred | COMP |
| RRRRNRTRRNRRRVRGC | [3] | C2Pred | COMP | PACGGFWISGRPG | [3] | C2Pred | COMP |
| AGYLLGKLKALAALAKKIL | [2] | CPPsite 2 | COMP | CLGSGEQCVRDTSCCSMSCTNNICF | [3] | C2Pred | COMP |
| RXVYXVLLSQ | [2] | CPPsite 2 | COMP | SYCGSTTRICCGYCAYFGKKCIDYPSN | [3] | C2Pred | COMP |
| LLIALRRRIRKQAXAXSK | [2] | CPPsite 2 | COMP | DCCPAKLLCCNP | [3] | C2Pred | COMP |
| RQIKIWFQNRRMKWKKLRKKKKKH | [3] | C2Pred | COMP | LPYPVNCKTECECVMCGLGIICKQCYYQQ | [3] | C2Pred | COMP |
| DTWAGVEAIIRILQQLLFIXFR | [2] | CPPsite 2 | COMP | IVAVLFLTACQFNAADDSRVRRNAEH | [3] | C2Pred | COMP |
| VSRRRRRRGGRRRRK | [2] | CPPsite 2 | COMP | GFMDTAKNVAKNVAVTLIDKLRCKVTGGC | [3] | C2Pred | COMP |
| LKKLAELAXKLLKLG | [2] | CPPsite 2 | COMP | SDLTWTYQSPGDPTNSKN | [3] | C2Pred | COMP |
| GRGDSPRRSPRR | [3] | C2Pred | COMP | GWMSKIASGIGTFLSGIGQQG | [3] | C2Pred | COMP |
| WLRRIKAWLRRIKALNRQLGVAA | [2] | CPPsite 2 | COMP | APEESPKRAPSGFLGVR | [3] | C2Pred | COMP |
| YGRRRRRRRRR | [2] | CPPsite 2 | COMP | KKAVRRQEAVDAL | [3] | C2Pred | COMP |
| YPYDANHTRSPT | [3] | C2Pred | COMP | GWKDWLNKGKEWLKKKGPGIMKAALKAATQ | [3] | C2Pred | COMP |
| GALFLGFLGAAGSTMGAWSQPKKKRKV | [2] | CPPsite 2 | COMP | MKLNGGKSLDPTGLY | [3] | C2Pred | COMP |
| CGGKDCERRFSRSDQLKRHQRRHTGVKPFQ | [3] | C2Pred | COMP | DDASDRAKKFYGLM | [3] | C2Pred | COMP |
| GWTLNSAGYLLGPXAVGNXRSFSDKNGLTS | [2] | CPPsite 2 | COMP | SSPETLISDLLMRESTENVPRTRLEDPAMW | [3] | C2Pred | COMP |
| SWLPYPWXVPSS | [2] | CPPsite 2 | COMP | YGGFMKPYTKQSHKPLITLLKHITLKNEQ | [3] | C2Pred | COMP |
| ACRGRGRRCGSGSRSCG | [2] | CPPsite 2 | COMP | TCCKFQFLNFCCNE | [3] | C2Pred | COMP |
| WIIFRIAAYXKK | [2] | CPPsite 2 | COMP | SAATNAVHRCCLTGCTQQDLLGLCPH | [3] | C2Pred | COMP |
| ARRRRCSDRFRNCPADEALCGRRRR | [2] | CPPsite 2 | COMP | VLSHNNESSYSDTSSCTSQ | [3] | C2Pred | COMP |
| GRQLRIAGKRLEGRSK | [2] | CPPsite 2 | COMP | VMMVEAGFGTHGCPLLQGTCDSHCRGMDA | [3] | C2Pred | COMP |
| RLLRLLLRLWRRLLRLLR | [3] | C2Pred | COMP | AADHDVGSELPPEGVLGALLRV | [3] | C2Pred | COMP |
| KLGVM | [2] | CPPsite 2 | COMP | RMTLSEKCCQVGCIRKDIARLC | [3] | C2Pred | COMP |
| KKTTTKPTKK | [2] | CPPsite 2 | COMP | KKDGYPVEYDRAY | [3] | C2Pred | COMP |
| CTWLKYX | [2] | CPPsite 2 | COMP | FLKPLFNAALKLLP | [3] | C2Pred | COMP |
| KHKLLHLLHLLALLWLHLLHLLKHK | [3] | C2Pred | COMP | GIGAILKVLATGLPTLISWIKNKRKQ | [3] | C2Pred | COMP |
| PSKRLLHNNLRR | [3] | C2Pred | COMP | ARHPHPHLSFM | [3] | C2Pred | COMP |
| MAARLCCQLDPARDV | [2] | CPPsite 2 | COMP | GRGARRYCGRVLADTLAYLCPEMEEVE | [3] | C2Pred | COMP |
| RGERLERRELRLERRELRC | [2] | CPPsite 2 | COMP | CKGKGAPCTRLMYDCCHGSCSSSKGRC | [3] | C2Pred | COMP |
| KRIIQRILSRNS | [3] | C2Pred | COMP | EEEESRPRKLCGRHLLIEVIKLCGQSDWS | [3] | C2Pred | COMP |
| RLLRLLRLL | [2] | CPPsite 2 | COMP | PPPPGGPQPRPPQG | [3] | C2Pred | COMP |
| RSVTTEINTLFQTLTSIAEKVDP | [3] | C2Pred | COMP | RCTCTTIISSSSTF | [3] | C2Pred | COMP |
| SMLKRNXSTSNR | [2] | CPPsite 2 | COMP | KPKCGLCRYRCCSGGCSSGKCVNGACDCS | [3] | C2Pred | COMP |
| RQPKIWFPNRRMPWKK | [3] | C2Pred | COMP | GIMDSVKGLAKNLAGKLLDSLKCKITGC | [3] | C2Pred | COMP |
| RQIKIWAQNRRMKWKK | [2] | CPPsite 2 | COMP | GLFLDTLKKFAKAGMEAVINPK | [3] | C2Pred | COMP |
| ARCSDRFRNCPADEALCGR | [2] | CPPsite 2 | COMP | DEATVFGLWPLCSYRMLPF | [3] | C2Pred | COMP |
| VELPPPVELPPPVELPPP | [3] | C2Pred | COMP | RSNKGFNFMVDMIQALSK | [3] | C2Pred | COMP |
| MDAQTRRRERRAEKQAQWKAANGC | [3] | C2Pred | COMP | GLISGLLGVGKMLVCGLSGLC | [3] | C2Pred | COMP |
| HPGSPFPPEHRP | [3] | C2Pred | COMP | LETPAPQVPARRLLPP | [3] | C2Pred | COMP |
| PPXNRIQRRLNM | [2] | CPPsite 2 | COMP | LLGMIPLAISAISALSKL | [3] | C2Pred | COMP |
| GPFXFYQFLFPPV | [2] | CPPsite 2 | COMP | DGKLYKMTHFRWSEGS | [3] | C2Pred | COMP |
| GDXLPXLKLC | [2] | CPPsite 2 | COMP | AKKELCTCQQPKHLKYIEKGLQKAKDYAT | [3] | C2Pred | COMP |
| RXNXRFNFRFFFNFRFNTRTN | [2] | CPPsite 2 | COMP | DSLSFSYNNFEEDD | [3] | C2Pred | COMP |
| LIRLWSXLIXIWFQNRRLKWKKK | [2] | CPPsite 2 | COMP | LAKRADICQPGKTSQRACET | [3] | C2Pred | COMP |
| KKKKKKNKKLQQRGD | [3] | C2Pred | COMP | GFFTLIKAANKLINKTVNKEAGKGGLEIMA | [3] | C2Pred | COMP |
| LIIFAILISXKK | [2] | CPPsite 2 | COMP | KCCMRPICMCPCCIGAG | [3] | C2Pred | COMP |
| INLKKLAKLXKKIL | [2] | CPPsite 2 | COMP | QPSYDRDIMSFG | [3] | C2Pred | COMP |
| GIGKFLXSAKKWGKAFVGQIMNC | [2] | CPPsite 2 | COMP | GLFDIVKKVVGTIAGLG | [3] | C2Pred | COMP |
| GKINLKALAALAKKIL | [2] | CPPsite 2 | COMP | IRDECCSNPACRVNNPHVC | [3] | C2Pred | COMP |
| RRRRRRRGGIYLATALAKWALKQ | [2] | CPPsite 2 | COMP | FFPMLAGVAARVVPKVICLITKKC | [3] | C2Pred | COMP |
| IPSRWKDQFWKRWXY | [2] | CPPsite 2 | COMP | FLPFIAGMAANFLPKIFCAISKKC | [3] | C2Pred | COMP |
| FITKALGISYGRKKRR | [2] | CPPsite 2 | COMP | WEAKLAKALAKALAKHLAKALKAKALCEA | [3] | C2Pred | COMP |
| MAIYRDLIS | [2] | CPPsite 2 | COMP | GFFDRIKALTKNVTLELLNTITCKLPVTPP | [3] | C2Pred | COMP |
| EEEAA | [2] | CPPsite 2 | COMP | AVLDFIKAAGKGLVTNIMEKVG | [3] | C2Pred | COMP |
| DRRRRGSRPSGAERRRRRAAAA | [3] | C2Pred | COMP | GMATKAGTALGKVAKAVIGAAL | [3] | C2Pred | COMP |
| RKKARQRRR | [2] | CPPsite 2 | COMP | WQIPEQSQCQAI | [3] | C2Pred | COMP |
| KMDSRWRWKSSKK | [2] | CPPsite 2 | COMP | YLRNPRKNLLKNILADVLARQLQKK | [3] | C2Pred | COMP |
| SHAFTWPTYLQL | [3] | C2Pred | COMP | YVSQRLCGSQLVDTLYSVCRHRGFYRPND | [3] | C2Pred | COMP |
| RQIKIWFQNRRMKWAK | [2] | CPPsite 2 | COMP | AGETHTVMINHAGRGAPKLVVGGKKLS | [3] | C2Pred | COMP |
| EKGKKIFIMK | [2] | CPPsite 2 | COMP | AFDSLAGSGFDNGFN | [3] | C2Pred | COMP |
| MDCRWRWKCCKK | [2] | CPPsite 2 | COMP | LEELEEELEGCE | [3] | C2Pred | COMP |
| GRKKRRQRPPQC | [2] | CPPsite 2 | COMP | YCNGKRVCVCRG | [3] | C2Pred | COMP |
| VPMIK | [2] | CPPsite 2 | COMP | NSELINSLLGIPKVMTDA | [3] | C2Pred | COMP |
| RQIKIAFQNRRMKWKK | [2] | CPPsite 2 | COMP | CFKKDMHKVETYL | [3] | C2Pred | COMP |
| EEEEEEEEPLGLAGRRRRRRRRN | [3] | C2Pred | COMP | FLGVVFKLASKVFPAVFGKV | [3] | C2Pred | COMP |
| FAPWDTASFMLG | [3] | C2Pred | COMP | INLKAIAALARNY | [3] | C2Pred | COMP |
| XYRIKPTFRRLKWKYKGKFA | [2] | CPPsite 2 | COMP | RGPDHRFAFGL | [3] | C2Pred | COMP |
| PQNRLQIRRXSK | [2] | CPPsite 2 | COMP | HADGRYTSDISSYLEGQAAKEFIAWLVNGR | [3] | C2Pred | COMP |
| MVKSKIGSWILVLFVAMWSDVGLCKKRPKP | [3] | C2Pred | COMP | FFPLLFGALSSHLPKLF | [3] | C2Pred | COMP |
| CRKKRRQRRR | [2] | CPPsite 2 | COMP | GRVRDQIMLSLGG | [3] | C2Pred | COMP |
| GWTLNPPGYLLGKINLKALAALAKKIL | [2] | CPPsite 2 | COMP | HIGPNPVYSAVSNTD | [3] | C2Pred | COMP |
| KCFQWQRNMRKVR | [2] | CPPsite 2 | COMP | DTNFPICLFCCKCCKNSSCGLCCIT | [3] | C2Pred | COMP |
| RSRGRLRRGAIRLQRG | [2] | CPPsite 2 | COMP | FPPPGESAVDMSFFYALSNP | [3] | C2Pred | COMP |
| KGKKIFIMK | [2] | CPPsite 2 | COMP | YGGFIGIRKSARKWNNQ | [3] | C2Pred | COMP |
| GRKKRRQRRRP | [2] | CPPsite 2 | COMP | DVDFNSESTRRKNKQKEIVDLHNSLKKTV | [3] | C2Pred | COMP |
| LLKTTELLKTTELLKTTE | [3] | C2Pred | COMP | ALWKDMLSGIGKLAGQAALGAVKTLV | [3] | C2Pred | COMP |
| VRLPPP | [2] | CPPsite 2 | COMP | RKFHEKHHSHRGYRSNYLYDN | [3] | C2Pred | COMP |
| RRRRRRRXXX | [2] | CPPsite 2 | COMP | YSSQHLCGSNLVEALYMTCGRSGFYRPHD | [3] | C2Pred | COMP |
| RKKRRQRRRGGGKLLKLLLKLLLKLLK | [3] | C2Pred | COMP | GIFSTVFKAGKGIVCGLTGLC | [3] | C2Pred | COMP |
| MIIYRDLISKK | [3] | C2Pred | COMP | RRKMCGEALIQALDVICVNGFT | [3] | C2Pred | COMP |
| KKWKMRRGAGRRRRRRRRR | [2] | CPPsite 2 | COMP | RPWCHPINAILAVEKVVCTYRDVRFESIRL | [3] | C2Pred | COMP |
| KALAKALAKLWKALAKAA | [2] | CPPsite 2 | COMP | ACAAHCLLRGNRGGYCNGKG | [3] | C2Pred | COMP |
| GWTLNSKINLKALAALAKKIL | [2] | CPPsite 2 | COMP | ALNSVAYERSVMQDYE | [3] | C2Pred | COMP |
| RQIKIWFQNRRAKWKK | [2] | CPPsite 2 | COMP | ILGTILGLLKGL | [3] | C2Pred | COMP |
| ACSGSGSGCGSGSGSCGRRRRRRRR | [2] | CPPsite 2 | COMP | SPVDYDRPIMAFG | [3] | C2Pred | COMP |
| ARRRCSGSGSGCGSGSGSCGRRR | [2] | CPPsite 2 | COMP | GLLGGLLGPLLGGGGGGGGGLL | [3] | C2Pred | COMP |
| RRGC | Oligoarginine R2 | CPPsite 2 | EXPR (PDB code: 3C88) | FLPGLIAGIAKML | [3] | C2Pred | COMP |
| MXKRPTTPSRKM | [2] | CPPsite 2 | COMP | ITCQQVTSELGPCVPYLTGQGIP | [3] | C2Pred | COMP |
| LLRILRRSIRRARRAIRR | [3] | C2Pred | COMP | RVCFAIPLPICH | [3] | C2Pred | COMP |
| KRIPNKKPGKKT | [2] | CPPsite 2 | COMP | MGMRLPNIIFL | [3] | C2Pred | COMP |
| GSRXPSLIIPRQ | [2] | CPPsite 2 | COMP | GGTYSCHFGPLTWVCKPQGG | [3] | C2Pred | COMP |
| GRKKRRQARAPPQC | [3] | C2Pred | COMP | VWPLGLVICKALKIC | [3] | C2Pred | COMP |
| WELYGRKKRRQRRR | [2] | CPPsite 2 | COMP | WLNALLHHGLNCAKGVLA | [3] | C2Pred | COMP |
| LAQLLAQLLAQLGGGGRRRRRRRRR | [2] | CPPsite 2 | COMP | FLPLAIGLLGKLFG | [3] | C2Pred | COMP |
| YSHIATLPFTPT | [3] | C2Pred | COMP | QWGYGGMPYGGYGGMGGYGMGGYGMGY | [3] | C2Pred | COMP |
| AAVALLPAVLLALLAPRRRRRR | [2] | CPPsite 2 | COMP | FLPLFLPKIICVITKKC | [3] | C2Pred | COMP |
| VGALAVVVWLWLWLWAGSGPKKKRKVC | [2] | CPPsite 2 | COMP | QGVNDNEEGFFSAR | [3] | C2Pred | COMP |
| RQIKIWFQNRAMKWKK | [2] | CPPsite 2 | COMP | NGARVSDMFRPSGDDFGDYSANWGDF | [3] | C2Pred | COMP |
| GCGGGYGRKKRRQRRR | [3] | C2Pred | COMP | GFLDKLKKGASDFANALVNSIKGT | [3] | C2Pred | COMP |
| RFTFHFRFEFTFHFEGGGRRRRRRR | [3] | C2Pred | COMP | KRGGAQYAPYWQETYLRSRK | [3] | C2Pred | COMP |
| GKKALKLAAKLLKKC | [3] | C2Pred | COMP | QWAQWPRPTPQIPP | [3] | C2Pred | COMP |
| RKKRRQRRA | [2] | CPPsite 2 | COMP | SLSRFLSFLKIVYPPAF | [3] | C2Pred | COMP |
| KPRSKNPPKKPK | [3] | C2Pred | COMP | VVNTPGHAVSYHVY | [3] | C2Pred | COMP |
| LLIILRRRIRKQAAAXSK | [2] | CPPsite 2 | COMP | IKIMDILAKLGKVLAHVG | [3] | C2Pred | COMP |
| LTMPSDLQPVLW | [3] | C2Pred | COMP | DVLKKIGTVALHAGKAALGAVADTISQ | [3] | C2Pred | COMP |
| RRRRRRRRRXXX | [2] | CPPsite 2 | COMP | FLSAITSLLGKLL | [3] | C2Pred | COMP |
| QIKIWFQNRRMKWKK | [2] | CPPsite 2 | COMP | SDEDSDGDRPQASPGLGPGP | [3] | C2Pred | COMP |
| KLALKALKAALKLA | [2] | CPPsite 2 | COMP | GIVEQCCDTPCSLYDPENYCN | [3] | C2Pred | COMP |
| RHNFRFFFNFRTNR | [3] | C2Pred | COMP | SGTGLSATLPQRF | [3] | C2Pred | COMP |
| MLLLTRRRST | [2] | CPPsite 2 | COMP | GTLPCESCVWIPCISSVVGCSCKSKVCYKN | [3] | C2Pred | COMP |
| WIIFRIAASXKK | [2] | CPPsite 2 | COMP | GCCSTPPCAVLYC | [3] | C2Pred | COMP |
| IRQRRRR | [2] | CPPsite 2 | COMP | VCIADDMPCGFGLFGGPLCCSGWCLFVCL | [3] | C2Pred | COMP |
| RQIKIWFQNRRMKWKA | [2] | CPPsite 2 | COMP | KGAAKGLLEVASCKLSKSC | [3] | C2Pred | COMP |
| CSKSSDYQC | [2] | CPPsite 2 | COMP | MRTWACLLLLGCGYLAFALAV | [3] | C2Pred | COMP |
| CGNKRTR | [2] | CPPsite 2 | COMP | SVLTPSLSSLGESLESGIS | [3] | C2Pred | COMP |
| SWLPYPWHVPSS | [3] | C2Pred | COMP | LNENLLRFFVAPFPEVFG | [3] | C2Pred | COMP |
| RQIKAWFQNRRMKWKK | [2] | CPPsite 2 | COMP | ALQTLPAMCNVY | [3] | C2Pred | COMP |
| GRRERNKMAAAKCRNRRR | [2] | CPPsite 2 | COMP | RDSLQRGGQKILEKAERIGDRIKDIFRG | [3] | C2Pred | COMP |
| RVTSWLGRQLRIAGKRLEGRSK | [2] | CPPsite 2 | COMP | TAEALRCQENYLPSPCQ | [3] | C2Pred | COMP |
| SWWTPWXVXSES | [2] | CPPsite 2 | COMP | FLPAVIRVAANVLPTAFCAISKKC | [3] | C2Pred | COMP |
| YARAARRAARR | [2] | CPPsite 2 | COMP | QCCITIPECCRI | [3] | C2Pred | COMP |
| RWRRWWRRW | [2] | CPPsite 2 | COMP | GCCSDPRCRYRC | [3] | C2Pred | COMP |
| GGRRARRRRRR | [2] | CPPsite 2 | COMP | QIDPLGFSGGI | [3] | C2Pred | COMP |
| XATKSQNINF | [2] | CPPsite 2 | COMP | NGGTSGLFAFPRV | [3] | C2Pred | COMP |
| WLKLWKKWLKLW | [2] | CPPsite 2 | COMP | GLFDIIKNIVSTL | [3] | C2Pred | COMP |
| RLPRPRPRPLPFPRPG | [2] | CPPsite 2 | COMP | FLAGLIGGLAKML | [3] | C2Pred | COMP |
| KRIPNKKPGKKTTTKPTKKPTIKTTKKDLK | [3] | C2Pred | COMP | INWLKLGKAIIDAL | [3] | C2Pred | COMP |
| RKKNPNCRRX | [2] | CPPsite 2 | COMP | FLPAVLRVAAKVGPAVFCAITQKC | [3] | C2Pred | COMP |
| WEAKLAKALAKALAKHLAKALAKALKACEA | [3] | C2Pred | COMP | ASEDALFGTMRF | [3] | C2Pred | COMP |
| SWAQXLSLPPVL | [2] | CPPsite 2 | COMP | SPMQRSSMVRF | [3] | C2Pred | COMP |
| GALFLGFLGAAGSTMGAWSQPKSKRKV | [3] | C2Pred | COMP | PFSLIPHAIGGLISAIK | [3] | C2Pred | COMP |
| RLLRLLRRLLRLLRRLLRC | [3] | C2Pred | COMP | SEAAALPRASAAAMRAAWPSPSVERV | [3] | C2Pred | COMP |
| RIFIRIGC | [2] | CPPsite 2 | COMP | VPSAGDMMVRF | [3] | C2Pred | COMP |
| RRRRRRRQIKILFQNRRMKWKKGGC | [3] | C2Pred | COMP | TQRLANFLIHSSNNFGAIFSPPN | [3] | C2Pred | COMP |
| CRGDC | [2] | CPPsite 2 | COMP | GKLQAFLAKMKEIAAQTL | [3] | C2Pred | COMP |
| XRXIRRQSLIML | [2] | CPPsite 2 | COMP | KLSPSLGPVSKGKLLAGQR | [3] | C2Pred | COMP |
| FQNRRMKWKK | [2] | CPPsite 2 | COMP | EWKLPDLIINHITLTRRNCNKYRCG | [3] | C2Pred | COMP |
| RKKRRQRRRGGG | [2] | CPPsite 2 | COMP | GFGMLFKFLAKKVAKKLVSHVAQKQLE | [3] | C2Pred | COMP |
| PIRRRKKLRRLK | [3] | C2Pred | COMP | CTCFTYKDKECVYYCHLDIIWINTP | [3] | C2Pred | COMP |
| VKRFKKFFRKLKKKV | [2] | CPPsite 2 | COMP | FLPFLIPALTSLISSL | [3] | C2Pred | COMP |
| RILQQLLFIXF | [2] | CPPsite 2 | COMP | SMAMGRLGLRPG | [3] | C2Pred | COMP |
| GSPWGLQHHPPRT | [3] | C2Pred | COMP | KLFNGNEVCLDPKEKWVQKVVQIFLK | [3] | C2Pred | COMP |
| MIIYRIAASXKK | [2] | CPPsite 2 | COMP | DCLPGWSVYEGRCYKVFNQKTWKAAEKFC | [3] | C2Pred | COMP |
| TRRSKRRSHRKF | [3] | C2Pred | COMP | MMRDSGCFGRRLDRIGSLSGLGCNVLRRY | [3] | C2Pred | COMP |
| AAVACRICMRNFSTRQARRNHRRRHRR | [3] | C2Pred | COMP | QEADPSSSLEADSTLKDEPRELSNM | [3] | C2Pred | COMP |
| CGGGRRRRRRRRRLLLL | [2] | CPPsite 2 | COMP | AGNLSECFWKYCV | [3] | C2Pred | COMP |
| WELVVLGKYGRKKRRQRRR | [2] | CPPsite 2 | COMP | GGAGEPLAFSPDMLSLRF | [3] | C2Pred | COMP |
| KWFKIQMQIRRWKNKR | [3] | C2Pred | COMP | GLEESPGHPGQPGPPGPPGAPGP | [3] | C2Pred | COMP |
| RWRCKNQN | [2] | CPPsite 2 | COMP | MYKIQLLSCIALTLALVANGAPTSSSTGNT | [3] | C2Pred | COMP |
| LNVPPSWFLSQR | [3] | C2Pred | COMP | FKAPYNIHWHCKPGLLC | [3] | C2Pred | COMP |
| YGRKKRRQRRRSVYDFFVWL | [3] | C2Pred | COMP | PAETPNSLDLTFNRRIMDTI | [3] | C2Pred | COMP |
| QRIRKSKISRTL | [3] | C2Pred | COMP | HSDAVFTDNYTRLRKQMAVKKYLNSILN | [3] | C2Pred | COMP |
| LCLR | [2] | CPPsite 2 | COMP | FSETIPAPTSKNEAQQKS | [3] | C2Pred | COMP |
| VPALR | [2] | CPPsite 2 | COMP | GPRPPGFSPFRGKFHSQS | [3] | C2Pred | COMP |
| GYGRKKRRQRRRG | [2] | CPPsite 2 | COMP | SDPSVPVEPEDDDMVDQ | [3] | C2Pred | COMP |
| PLSSIFSRIGDP | [3] | C2Pred | COMP | MIASHLAFEKLSKLGSKHTML | [3] | C2Pred | COMP |
| RQIRIWFQNRRMRWRRC | [3] | C2Pred | COMP | RSLDASPSSAFSGNHSLS | [3] | C2Pred | COMP |
| RQIKIWFQNRRMAWKK | [2] | CPPsite 2 | COMP | SEAAALPRASAAAMSCVAEPECREG | [3] | C2Pred | COMP |
| LCL | [2] | CPPsite 2 | COMP | SSSMYDRDIMSFG | [3] | C2Pred | COMP |
| KMDCRWRWKSSKK | [2] | CPPsite 2 | COMP | MKVFFLFAVLFCLVRRNSVHISHQEARGP | [3] | C2Pred | COMP |
| SRRXXCRSKAARSRXX | [2] | CPPsite 2 | COMP | SVNTKNDFMRF | [3] | C2Pred | COMP |
| RTLVNEYKNTLKFSK | [3] | C2Pred | COMP | MQFITDLIKKAVDFFKGLFGNK | [3] | C2Pred | COMP |
| YARKARRAARR | [2] | CPPsite 2 | COMP | SWPVCTRNGLPVCGETCVGGTCNTPGCTC | [3] | C2Pred | COMP |
| LLIILRRRARKQAXAXSK | [2] | CPPsite 2 | COMP | MSNRGASLKGLFLAVLLVSNTLLTKEGVT | [3] | C2Pred | COMP |
| CIGAVLKVLTTGLPALISWIKRKRQQ | [3] | C2Pred | COMP | GWFDVVKHIASAV | [3] | C2Pred | COMP |
| WKCRRQCFRVLXXWN | [2] | CPPsite 2 | COMP | STDCGGPKTQPLACDHPPLPDILFL | [3] | C2Pred | COMP |

Table S5: Sequences of CPPs and non-CPPs used in the training dataset with their origin (CPPSite 2, C2Pred, or DB1*) and their modelling origin: experimental (EXPR) or computational (COMP).

* DB1 means that peptides were obtained from Sanders *et al* (2011) [1].

| **Sequence** | **Reference** | **Origin** | **Model** | **Sequence** | **Reference** | **Origin** | **Model** |
| --- | --- | --- | --- | --- | --- | --- | --- |
| KWRRKLKKLRPKKKRKV | LDP-NLS | DB2 | COMP | KKLSECLKRIGDELDS | Bax BH3 | DB1 | COMP |
| KALKKLLAKWAAAKALL | MAP 8 | DB1 | COMP | RPPGFSPFR | Bradykinin | PDB | EXPR (PDB code: 6F3V) |
| RRLSSYSSRRRF | SynB3 | DB1 | COMP | IAARIKLRSRQHIKLRHL | scr pVEC | DB1 | COMP |
| GRKKRRQRRRPPC | ptat4 | DB1 | COMP | CYFQNCPRG | Vasopressin | DB1 | COMP |
| CNGRCG | Aminopeptase | DB1 | COMP | FVPIFTHSELQKIREKERNKGQ | Motolin | DB1 | COMP |
| LIRLWSHLIHIWFQNRRLKWKKK | EB1 | DB1 | COMP | AWRRKLKALAPAKKAKV | Mut-LDP-NLS | DB2 | COMP |
| AHALCPPERQIKIWFQNRRMKWKKEN | pAntpHD 40P2 | DB1 | COMP | KIWFQNRRMK | pAntp(4-13) | DB1 | COMP |
| AAVALLPAVLLALLAKNNLKDCGLF | [1] | DB1 | COMP | DSSNLPPNQKQIVD | [3] | C2Pred | COMP |
| CNGRCGGKLAKLAKLAKLAK | [1] | DB1 | COMP | VKRCCDEEECSSACWPCCWG | [3] | C2Pred | COMP |
| GGRQIKIWFQNRRMKWKK | [1] | DB1 | COMP | LLKELWTKMKGAGKAVLGKIKGLL | [3] | C2Pred | COMP |
| LLIILRARIRKQAHAHSK | [1] | DB1 | COMP | TTITVVNKCSYTVWPGALPGGGVVLD | [3] | C2Pred | COMP |
| MDAQTRRRERRAEKQAQWKAAN | [1] | DB1 | COMP | YKVDEDLQGAGGIQSRGYFFFRPRN | [3] | C2Pred | COMP |
| MGLGLHLLVLAAALQGAKKKRKV | [1] | DB1 | COMP | DHLPHDVYSPRL | [3] | C2Pred | COMP |
| NAKTRRHERRRKLAIER | [1] | DB1 | COMP | APGDRIYVHPF | [3] | C2Pred | COMP |
| CGRKKRWWRQRRRPPQ | [2] | CPPsite 2 | COMP (CPPSite ID: 2623) | HADGLFTSGYSKLLGQLSARRYLESLI | [3] | C2Pred | COMP |
| MIIYRDL | TCTP (1-7) | CPPsite 2 | COMP (CPPSite ID: 1586) | EGGGPQWAVGHFM | [3] | C2Pred | COMP |
| LLRARWRRRRSRRFR | [3] | C2Pred | COMP | MHVERRECAYCLTINTTICAGYCMTR | [3] | C2Pred | COMP |
| GGGRRRRRRYGRKKRRQRR | [3] | C2Pred | COMP | AALKGCWTKSIPPKPCSGKR | [3] | C2Pred | COMP |
| GRQLRRAGRRLRGRSR | [3] | C2Pred | COMP | GVVTDLLKTAGKLLGNLVGSLSG | [3] | C2Pred | COMP |
| YRRAARRAARA | CTP503 | CPPsite 2 | COMP (CPPSite ID: 1724) | GWASSIGSILGKFAKGGAQAFLQPK | [3] | C2Pred | COMP |
| GWTLNSAGYLLGKINLKALAALAKKLL | TP2 | CPPsite 2 | COMP (CPPSite ID: 1045) | SYGWAEGDTTDNEYLRF | [3] | C2Pred | COMP |
| GSVSRRRRRRGGRRRR | [3] | C2Pred | COMP | GFGSFLGKALKAALKIGANVLGGAPQQ | [3] | C2Pred | COMP |
| IIYRDLISX | [2] | CPPsite 2 | COMP | AIFIFIRWLLKLGHHGRAPP | [3] | C2Pred | COMP |
| CGYGRKKRRQRRRGC | Tat | CPPsite 2 | COMP (CPPSite ID: 2491) | GCCSHPACNVNNPHICG | [3] | C2Pred | COMP |
| RXXLRXLRRXL | F3 | CPPsite 2 | COMP (CPPSite ID: 2924) | GNNRPVYIPQPRPPHPRI | [3] | C2Pred | COMP |
| VCVR | [2] | CPPsite 2 | COMP | DTHISEKIIDCNDIG | [3] | C2Pred | COMP |
| RKSSKPIMEKRRRAR | [3] | C2Pred | COMP | MWITNGGVANWYFVLAR | [3] | C2Pred | COMP |
| KFFKFFKFFK | CPP-PNA | CPPsite 2 | COMP (CPPSite ID: 2072) | QGLPPGPPIPR | [3] | C2Pred | COMP |
| KETWFETWFTEWSQPKKKRKV | [2] | CPPsite 2 | COMP | NPKVAHCASQIGRSTAWGAVSGA | [3] | C2Pred | COMP |
| KALAALLKKLAKLLAALK | [2] | CPPsite 2 | COMP | RCCQTFYWCCVQ | [3] | C2Pred | COMP |
| WELVYGRKKRRQRRR | [2] | CPPsite 2 | COMP | ADDSDPVGGEFLAEGGGVR | [3] | C2Pred | COMP |
| LCLK | [2] | CPPsite 2 | COMP | DNTVTSKPLNCMNYFWKSRTAC | [3] | C2Pred | COMP |
| GLFKALLKLLKSLWKLLLKA | [2] | CPPsite 2 | COMP | DGCSNAGAFCGIHPGLCCSEICIVWCT | [3] | C2Pred | COMP |
| KMIFVGIKKKEERA | [2] | CPPsite 2 | COMP | MKVSAAALAVILIATALCA | [3] | C2Pred | COMP |
| KRIPNKKPGKK | [2] | CPPsite 2 | COMP | SDPSVPLRPEEDELIDQ | [3] | C2Pred | COMP |
| GYGRKKRRGRRRTHRLPRRRRRR | [3] | C2Pred | COMP | CKGKGQSCSKLMYDCCTGSCSRRGKC | [3] | C2Pred | COMP |
| MVRRFLVTLRIRRACGPPRVRV | [3] | C2Pred | COMP | GVSFHPRLKEKDDNSSGNSRKSNPK | [3] | C2Pred | COMP |
| KRIHPRLTRSIR | [3] | C2Pred | COMP | IIPLPLGYFAKKT | [3] | C2Pred | COMP |
| NTCTWLKYX | [2] | CPPsite 2 | COMP | QADPNKFYGLM | [3] | C2Pred | COMP |
| RLWMRWYSPRTRAYG | [2] | CPPsite 2 | COMP | FLGRVLPPTRATASTHRSRL | [3] | C2Pred | COMP |
| ACSSSPSKXCG | [2] | CPPsite 2 | COMP | FWGHIWNAVKRVGANALHGAVTGALS | [3] | C2Pred | COMP |
| SARXXCRSKAKRSRXX | [2] | CPPsite 2 | COMP | GNTKKAVPGFYGTR | [3] | C2Pred | COMP |
| WEYGRKKRRQRRR | [2] | CPPsite 2 | COMP | FLPLVTMLLGKLF | [3] | C2Pred | COMP |
| FQWQRNMRKVRGPPVS | [2] | CPPsite 2 | COMP | VTMVEAGFGCPSFPSPRDSHCRGMGR | [3] | C2Pred | COMP |
| KRIXPRLTRSIR | [2] | CPPsite 2 | COMP | YGGFLRRIRFARKLANQ | [3] | C2Pred | COMP |
| RLVMRVYSPTTRRYG | [2] | CPPsite 2 | COMP | GNGVVLTLTHECNLATWTKKLKCC | [3] | C2Pred | COMP |
| KLALKLALKALKAA | [2] | CPPsite 2 | COMP | TMKLCGRKLPETLSKLCVY | [3] | C2Pred | COMP |
| RWRRWRRWRRWR | [3] | C2Pred | COMP | GLRSKIWLWVLLMIWQESNKFKKM | [3] | C2Pred | COMP |
| ACSDRFRNCPADEALCGRRRRRRRR | [2] | CPPsite 2 | COMP | KFDMVAYVSEEDS | [3] | C2Pred | COMP |
| LKTLTETLKELTKTLTEL | [3] | C2Pred | COMP | GLWSKIKDVAAAAGKAALGAVNEALGEQ | [3] | C2Pred | COMP |
| LGLLLRXLRXXSNLLANI | [2] | CPPsite 2 | COMP | SDRPTRAMDSPLIRF | [3] | C2Pred | COMP |
| KSHAHAQKRIRRRLIILL | [3] | C2Pred | COMP | QRFSQPTFKLPQGRLTLSRKF | [3] | C2Pred | COMP |
| GRKKRRQRARPPQC | [2] | CPPsite 2 | COMP | NGVCCGYKLCHPC | [3] | C2Pred | COMP |
| MRRIRPRPPRLPRPRPRPLPFPRPGGCYPG | [3] | C2Pred | COMP | LFAKINGLKVGPLKIQIV | [3] | C2Pred | COMP |
| PPRLPRPRPRPLPFPRPG | [2] | CPPsite 2 | COMP | NPELYQMNHFRWGQPPTHFKQ | [3] | C2Pred | COMP |
| RLYMRYYSPTTRRYG | [3] | C2Pred | COMP | GCCGSFACRFGCVPCCV | [3] | C2Pred | COMP |
| RRHLRRHLRHLRRHLRRHLRHL | [3] | C2Pred | COMP | FIITGLVRGLTKLF | [3] | C2Pred | COMP |
| CKYGRKKRRQRRR | [2] | CPPsite 2 | COMP | GSSFLSPEFKKIQQQNDPTKTTAKIH | [3] | C2Pred | COMP |
| CXAIYPRX | [2] | CPPsite 2 | COMP | IIDYYDEGEEDRDVGVVDAR | [3] | C2Pred | COMP |
| SRRXXCRAKAKRSRXX | [2] | CPPsite 2 | COMP | PLVQQQFLGQQQPFPPQ | [3] | C2Pred | COMP |
| GNYAHRVGAGAPVWL | [3] | C2Pred | COMP | NPFKELERAGQRVRDAIIS | [3] | C2Pred | COMP |
| YTFGLKTSFNVQYTFGLKTSFNVQ | [3] | C2Pred | COMP | CHRRDSHKIDNYFKVLKCRLIHDSNC | [3] | C2Pred | COMP |
| KRPAATKKAGQAKKKKL | [3] | C2Pred | COMP | ALFEESTVSAEPR | [3] | C2Pred | COMP |
| GRKRKKRT | [2] | CPPsite 2 | COMP | GIGGKPVQTAFVDNDGIYD | [3] | C2Pred | COMP |
| MIIYRDLI | [2] | CPPsite 2 | COMP | GAFGDLLKGVAKEAGLKLLNMAQCKLSGNC | [3] | C2Pred | COMP |
| GRRHHCRSKAKRSRHH | [3] | C2Pred | COMP | FFGHLYRGITSVVKHVHGLLSG | [3] | C2Pred | COMP |
| RKKRRRESWVXLPPPVXLPPPGGXXXXXX | [2] | CPPsite 2 | COMP | LRTLLELARTQSQRERAEQNRIIFDSVGK | [3] | C2Pred | COMP |
| RQARRNRRRALWKTLLKKVLKA | [3] | C2Pred | COMP | GLLSGVLGVGKKVDCGLSGLC | [3] | C2Pred | COMP |
| WELVVLYGRKKRRQRRR | [2] | CPPsite 2 | COMP | SLSYEDKMFDNVEFTPRL | [3] | C2Pred | COMP |
| RRRRRRRGGIYLATALAKWALKQGF | [3] | C2Pred | COMP | INWKAIIEAAKQAL | [3] | C2Pred | COMP |
| KLALKLALKWAKLALKAA | [3] | C2Pred | COMP | FLGALFKVASKVLPSVFCAITKKC | [3] | C2Pred | COMP |
| TKRRITPKDVIDV | [2] | CPPsite 2 | COMP | DGSVDFKKNWIQYKEGFGHLSPTG | [3] | C2Pred | COMP |
| RVRVFVVHIPRLT | [3] | C2Pred | COMP | NWTPQAMLYLKGAQ | [3] | C2Pred | COMP |
| WRFKWRFKWRFK | [3] | C2Pred | COMP | RELEELNVPGEIVESLSSSEESITRINK | [3] | C2Pred | COMP |
| RRGRRG | [2] | CPPsite 2 | COMP | MTDMWSLKICAWLGFLLLFKP | [3] | C2Pred | COMP |

Table S6: Sequences of CPPs and non-CPPs used in the independent test dataset with their origin (CPPSite 2, C2Pred, DB1*, or DB2^**^), and their model: Experimental (EXPR) or Computational (COMP).

* DB1 means the database from Sanders *et al* (2011) [1].

^**^ DB2 means the database from Ponnappan, N. & Chugh, A. (2017) [4].

**References**

[1] – Sanders, W. S., Johnston, C. I., Bridges, S. M., Burgess, S. C. & Willeford, K. O. Prediction of Cell Penetrating Peptides by Support Vector Machines. *PLoS Comput. Biol.* **7**, e1002101 (2011).

[2] – Agrawal, P. *et al.* CPPsite 2.0: a repository of experimentally validated cell-penetrating peptides. *Nucleic Acids Res.* **44**, D1098–D1103 (2016).

[3] – Tang, H., Su, Z.-D., Wei, H.-H., Chen, W. & Lin, H. Prediction of cell-penetrating peptides with feature selection techniques. *Biochem. Biophys. Res. Commun.* **477**, 150–154 (2016).

[4] - Ponnappan, N. & Chugh, A. Cell-penetrating and cargo-delivery ability of a spider toxin-derived peptide in mammalian cells. *European Journal of Pharmaceutics and Biopharmaceutics* 114, 145–153 (2017)
